# New bacterial strains for ibuprofen biodegradation: Drug removal, transformation, and potential catabolic genes

**DOI:** 10.1111/1758-2229.13320

**Published:** 2024-08-26

**Authors:** Alba Lara‐Moreno, Maria Clara Costa, Ayleen Vargas‐Villagomez, Jorge Dias Carlier

**Affiliations:** ^1^ Centre of Marine Sciences (CCMAR/CIMAR LA) University of the Algarve, Gambelas Campus Faro Portugal; ^2^ Department of Microbiology and Parasitology, Faculty of Pharmacy University of Seville Seville Spain; ^3^ Faculty of Sciences and Technologies University of the Algarve, Gambelas Campus Faro Portugal

## Abstract

Ibuprofen (IBU) is a significant contaminant frequently found in wastewater treatment plants due to its widespread use and limited removal during treatment processes. This leads to its discharge into the environment, causing considerable environmental concerns. The use of microorganisms has recently been recognized as a sustainable method for mitigating IBU contamination in wastewater. In this study, new bacteria capable of growing in a solid medium with IBU as the only carbon source and removing IBU from a liquid medium were isolated from environmental samples, including soil, marine, mine, and olive mill wastewater. Four bacterial strains, namely *Klebsiella pneumoniae* TIBU2.1, *Klebsiella variicola* LOIBU1.1, *Pseudomonas aeruginosa* LOIBU1.2, and *Mycolicibacterium aubagnense* HPB1.1, were identified through 16S rRNA gene sequencing. These strains demonstrated significant IBU removal efficiencies, ranging from 60 to 100% within 14 days, starting from an initial IBU concentration of 5 mg per litre. These bacteria have not been previously reported in the literature as IBU degraders, making this work a valuable contribution to further studies in the field of bioremediation in environments contaminated by IBU. Based on the IBU removal results, the most promising bacteria, *K. pneumoniae* TIBU2.1 and *M. aubagnense* HPB1.1, were selected for an in silico analysis to identify genes potentially involved in IBU biodegradation. Interestingly, in the tests with TIBU2.1, a peak of IBU transformation product(s) was detected by high‐performance liquid chromatography, while in the tests with HPB1.1, it was not detected. The emerging peak was analysed by liquid chromatography–mass spectrometry, indicating the presence of possible conjugates between intermediates of IBU biodegradation. The proteins encoded on their whole‐genome sequences were aligned with proteins involved in an IBU‐degrading pathway reported in bacteria with respective catabolic genes. The analysis indicated that strain HPB1.1 possesses genes encoding proteins similar to most enzymes reported associated with the IBU metabolic pathways used as reference bacteria, while strain TIBU2.1 has genes encoding proteins similar to enzymes involved in both the upper and the lower part of that pathway. Notably, in the tests with the strain having more candidate genes encoding IBU‐catabolic enzymes, no IBU transformation products were detected, while in the tests with the strain having fewer of these genes, detection occurred.

## Graphical abstract

This study explores the use of bacteria to mitigate ibuprofen (IBU) contamination in wastewater. Four new bacterial strains were identified that can grow with IBU as the only carbon source and demonstrated significant IBU removal efficiencies. In‐depth analysis revealed that two strains, *Klebsiella pneumoniae* TIBU2.1 and *Mycolicibacterium aubagnense* HPB1.1, possess genes encoding proteins similar to enzymes involved in IBU degradation, providing a promising avenue for bioremediation in IBU‐contaminated environments.

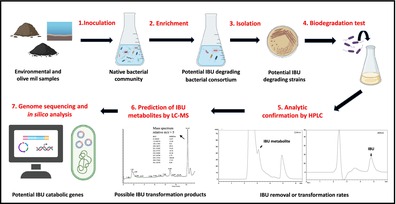

## INTRODUCTION

Wastewater treatment plants (WWTPs) with conventional treatments (primary and secondary) are not designed to eliminate pharmaceuticals (PCs), thereby allowing the release of the initial compounds or by‐products into the environment (Mezzelani et al., 2018; Sharma et al., 2019). Despite bringing countless benefits to society, these ubiquitous drugs represent an ecological risk when they are not eliminated from wastewater and make their way into aquatic environments (González Peña et al., 2021). The continuous discharge of these compounds, even at low concentrations ranging, from ng L^−1^ to μg L^−1^, can affect the environment and the health of aquatic and terrestrial organisms, leading to adverse effects. This is because PCs are designed to be biologically active at very low doses (Hodkovicova et al., 2022; Silva et al., 2021; Zhang et al., 2013). Based on the risk they pose, PCs were regarded as emerging water pollutants (Samal et al., 2022). Despite being considered emerging contaminants of concern and consistently being detected in wastewater, as indicated by the European Union's Environmental Quality Standards Directive 2013/39/EU (which is still in effect), there are currently no legal requirements for treating effluents contaminated with PC compounds. However, ibuprofen (IBU) is one of the compounds proposed as option 1 for addition to the list of individual priority substances in the EU proposal for a new Directive amending (Document 52022PC0540, 2022). The inclusion of IBU in the priority list signifies a positive step towards increased monitoring and control of this contaminant, since is one of the most frequently detected in surface waters, groundwater, and oceans worldwide (OECD, 2019). A study carried out in Portugal revealed that in the Faro region (south Portugal), the second most frequently detected compound in urban wastewater influent was IBU with a concentration of 13.3 μg L^−1^ (Silva et al., 2021). Despite IBU and its metabolite compounds being removed during the activated sludge process in WWTPs with an efficiency of 90–99%, it continues to be found in WWTP effluents, surface water, and non‐target organisms due to its high usage and its continuous release in the environment (Ferrando‐Climent et al., 2012; Larsson et al., 2014; Lee et al., 2020). This leads to IBU accumulation and the consequent emergence of detrimental on aquatic ecosystems. Furthermore, when this contaminant enters the food chain in a continuing and long‐term way, it poses a risk to human health. Therefore, it is necessary to regulate the discharge and spread, as well as to implement additional treatment methods in WWTPs to reduce the dispersion of IBU and its metabolites into the environment. Bioremediation techniques have gained attention for the removal of PCs in WWTPs, primarily due to their ability to produce non‐toxic by‐products alongside their impressive removal capacity (Show et al., 2022). Certain microorganisms such as bacteria, algae, and fungi possess the metabolic capacity to transform complex chemicals into simpler ones (metabolites) or achieve complete degradation, mineralizing the drug to carbon dioxide and water (Nyirenda et al., 2020). Sometimes the endogenous microbiota of the conventional activated sludge may not be effective in removing persistent organic pollutants. Therefore, it would be necessary to look for adapted microorganisms with the ability to degrade these compounds through enrichment cultures using the target contaminants as a carbon and energy source (Park & Oh, 2020a, 2020b). Despite the time and complexity required to isolate a microorganism or microbial consortium with the ability to degrade environmentally harmful pollutants like PCs, biodegradation is one of the most interesting strategies. Biodegradation is considered a cost‐effective, and environmentally friendly strategy (Aguilar‐Romero et al., 2022), especially when compared to other methods that generate harmful by‐products requiring treatment and having high operating costs, such as nanofiltration membranes (Maryam et al., 2020), electrochemical techniques (Nabgan et al., 2022), advanced oxidation processes (Mahbub & Duke, 2023), among others. The utilization of bioaugmentation strategies has demonstrated significant potential, enabling the scientific manipulation of sludge microenvironments to boost the populations of multiple bacteria with similar capabilities (Figdore et al., 2018). Various research studies have investigated the introduction of bacterial species into wastewater treatment bioreactors to enhance the PCs biodegradation (Bessa et al., 2021; Ely et al., 2022). These studies have expanded the prospects of bioaugmentation in WWTPs by offering a range of microorganism choices to complement the in situ microbial communities within the sludge (Duque et al., 2011). On the other hand, researchers can identify metabolic pathways involved in the degradation of IBU using bacteria that possess the ability to biodegrade this compound. Then, the understanding of IBU biodegradation pathways may lead to the development of more effective bioremediation strategies. To date, several pathways for the degradation of IBU have been elucidated in bacteria and key intermediate metabolites have been identified. For example, Chen and Rosazza (1994) investigated the microbial biotransformation of IBU by *Nocardia* sp. NRRL 5646, and identified two intermediates produced from this PC: ibuprofenol (MW ~192 g/mol) and IBU acetate (MW ~264 g/mol). In another study with *Sphingomonas* sp. Ibu‐2, it was proposed a biodegradation mechanism involving catechols as key intermediates, which are cleaved by dioxygenases to form compounds without aromatic rings (Murdoch & Hay, 2005, 2013). The authors suggested that Ibu‐2 metabolizes IBU through a mechanism resulting in the removal of the propanoic acid moiety and dioxygenation of the ring at the 1,2 position, giving rise to isobutylcatechol (MW ~166), which is further metabolized via meta‐cleavage. Indeed, it is now considered that a key intermediate in IBU biodegradation is isobutylcatechol, which is cleaved by a catechol 2.3‐dioxygenase and transformed into 5‐formyl‐2‐hydroxy‐7‐methylocta‐2,4‐dienoic acid (MW ~198 g/mol) (or 5‐formyl‐2‐hydroxy‐7‐methylocta‐2,4‐dienoate [MW ~197 g/mol]) (Aulestia et al., 2022). Then, Marchlewicz et al. (2017) identified a different IBU biodegradation pathway in *Bacillus thuringiensis* B1, highlighting the hydroxylation of both the aromatic ring and aliphatic chain as key steps. They found intermediates such as 2‐hydroxyibuprofen (MW ~222), 2‐(4‐hydroxyphenyl)propanoic acid (MW ~166 g/mol), 1,4‐hydroquinone (MW ~110 g/mol), and 2‐hydroxyquinol (or 2‐hydroxy‐1,4‐benzoquinone [MW ~124 g/mol]). Furthermore, Salgado et al. (2020) reported the biodegradation of IBU by the bacterium *Patulibacter medicamentivorans* under aerobic conditions and proposed two possible metabolic pathways for this drug based on the detected metabolites. The first involves the formation of Isobutylbenzene (~134) and 2‐phenylpropanoic acid (MW ~150) followed by gain of hydroxyl groups and formation of the intermediate catechols 3‐Isobutylbenzene‐x,ydiol (MW ~166) (such as 3‐Isobutylbenzene‐1,2diol or Isobutylcatechol) and 2‐(x,y‐dihydroxyphenyl)propanoic acid (MW ~183), and then the cleavage of aromatic rings and transformation into 5‐formyl‐2‐hydroxy‐7‐methylocta‐2,4‐dienoate (MW ~197 g/mol) and 6‐carboxy‐5‐formyl‐2,6‐dihydroxyhepta‐2,4‐dienoate (MW ~229), respectively, followed by their breakdown into smaller molecules, and eventual mineralization. The second mechanism proposed by Salgado et al. (2020) entails the hydroxylation and the carboxylation of the IBU molecule into compounds with higher masses, followed by several transformations that end in 3‐methoxy‐2‐(4‐(3‐methoxy‐2‐(methoxycarbonyl)‐3‐oxopropyl)phenyl)‐3‐oxopropanoic acid (MW ~338) and dimethyl 2‐(dihydroxy(4‐(1‐methoxy‐1‐oxopropan‐2‐yl)phenyl)methyl)malonate (MW ~340). Likewise, in a study conducted by Lu et al. (2019), metabolites also with higher masses than IBU were detected when *Pseudoxanthomonas* sp.DIN‐3 was inoculated: carboxyibuprofen 3‐(4‐(1‐carboxyethyl)phenyl‐2‐methylpropanoic acid) (MW ~236); hydroxyibuprofen 2‐(4‐(1‐hydroxy‐2‐methylpropyl)phenyl)propanoic acid (MW ~222); 3‐hydroxy‐2‐(4‐(1,1,2,3‐tetrahydroxy‐2‐(hydroxymethyl)propyl)phenyl)propanoic acid (MW ~302); 2‐(4‐(1,1‐dihydroxy‐2‐methylpropyl)phenyl)propanoic acid (MW ~238); 2‐(4‐(2‐formyl‐1,1‐dihydroxy‐3‐oxopropyl)phenyl)propanoic acid (MW ~266); 2‐((4‐(1‐carboxyethyl)phenyl)dihydroxymethyl)malonic acid (MW ~298) and dimethyl 2‐(dihydroxy(4‐(1‐methoxy‐1‐oxopropan‐2‐yl)phenyl)methyl)malonate (MW ~340).

In short, IBU can be biodegraded via the formation of compounds with smaller masses such as isobutylbenzene (~134) and 2‐phenylpropanoic acid (MW ~150), followed by gain of hydroxyl groups and the formation of 3‐Isobutylbenzene‐x,ydiol (such Isobutylcatechol) (MW ~166) and/or 2‐(x,y‐dihydroxyphenyl)propanoic acid (MW ~183), and then the cleavage of aromatic rings and transformation into 5‐formyl‐2‐hydroxy‐7‐methylocta‐2,4‐dienoate (MW ~197 g/mol) and/or 6‐carboxy‐5‐formyl‐2,6‐dihydroxyhepta‐2,4‐dienoate (MW ~229), respectively, but IBU can also undergo a series of metabolic and/or physicochemical transformations, including hydroxylation and carboxylation, that generate intermediate transformation products with masses much higher than IBU. Thus, typically, the initial step in IBU biodegradation involves the hydroxylation of either the aliphatic chain or the aromatic ring (Murdoch & Hay, 2005), and the *ipf* cluster, initially characterized in the *Sphingomonas* ibu‐2 strain by Murdoch and Hay (2005, 2013), is widely recognized as being involved in the initial steps of IBU biodegradation. Moreover, Aulestia et al. (2022) studied the IBU metabolic pathway of *Rhizorhabdus wittichii* MPO218, and in addition to the *ipf* genes previously described for the upper IBU biodegradation pathway, they have identified new genes required for the lower part of the metabolic pathway, thus proposing a putative complete pathway in that strain.

In the present study, 11 colony strains isolated in selective solid medium from enriched cultures grown from environmental samples potentially contaminated with aromatic compounds and from a sample of industrial olive mill wastewater, plus a bacterial strain isolated from a mine cave, were tested for the capacity to remove or transform IBU in an aqueous solution. Afterwards, the two most promising (*Mycolicibacterium aubagnense* HPB1.1 and *K. pneumonia* TIBU2.1) were studied at the genomic level to identify putative IBU‐degrading genes. These two strains were sent to a public collection and might be selected for further studies on biodegradation capacity, or to be directly used in projects on biotreatment improvement and/or as targets of study to increase and deepen knowledge about the genomic and functional background existing in bacteria aiming to enhance the development of genetic‐based tools and strategies to improve biotreatment processes.

## EXPERIMENTAL PROCEDURES

### Sample collection

The first thoughts on sampling for this type of work are to look at hospital effluents or urban waste discharges, and that is why usually that is the strategy followed. In this work, a different strategy was followed because the goal was to find species different from those already reported in the literature for IBU biodegradation. The samples used in this work to obtain IBU‐degrading microorganisms were collected in August and September of 2022 from different points in the Algarve area potentially exposed to aromatic hydrocarbons: 1. Tavira, a surface sample layer of marine sediments taken close to a boat fuel stand (37°07′28.8″N 7°38′38.4″W), 2. Santa Luzia, a sample of sandy soil contaminated with gasoline and oils in a confined boat maintenance area on the banks of the Ria Formosa estuary area (37°06′08.4″N 7°39′21.4″W), 3. Fuseta, a marine sample collected in a maritime port next to the municipal market (37°03′21.1″N 7°44′39.9″W), and 4. Santa Catarina da Fonte do Bispo, a sample from a pound of olive mill wastewater and its sediments (37°08′59.2″N 7°47′14.0″W). Samples were stored in plastic bottles at room temperature until the enrichment culture was performed.

### Bacterial strains

The strategy to increase the probability of isolating IBU‐degrading bacteria was based on the combination of three conditions: (i) the strains could prevail for a long period in enrichment cultures in a liquid medium in which the only carbon source added was IBU; (ii) the strains were capable of growing and forming colonies on a solid medium with ultrapure agar in which the only added carbon source was IBU; and (iii) the strains could remove IBU from liquid media and caused the appearance of transformation products. The methodologies underlying the implementation of this strategy are described below in the following sections.

Three new bacterial strains isolated in this study (*Klebsiella pneumoniae* TIBU2.1, *Klebsiella variicola* LOIBU1.1, and *Pseudomonas aeruginosa* LOIBU1.2) through enrichment cultures in the presence of IBU as outlined below, prevailed and have proliferated in solid medium when this drug was the only carbon source added, and removed IBU from solution. Moreover, one strain able to degrade hydroquinone (*Mycolicibacterium aubagnense* strain HPB1.1) previously isolated from a sample of wall moonmilk taken at Poderosa mine was also tested successfully for IBU removal from solution. These isolated bacteria were considered possible IBU biodegrading strains and have been preserved for further studies in cryovials at −20°C and −80°C with LB grow medium and 40% glycerol.

### Culture media

Lysogeny Broth (LB Broth) medium (Nzytech, Portugal) was prepared according to the supplier's instructions and used for the cultivation of bacterial strains. In the case of HPB1.1, the LB medium was supplemented with 5 ml L^−1^ of 99% glycerol (Fisher Chemical, Ref. G/0650/15, LOT: 1926887) to facilitate bacterial growth according to recommendations found in the BacDive database (Reimer et al., 2022). Finally, the mineral salt medium (MSM) base described by Zhang et al. (2013) supplemented with (NH_4_)_2_HPO_4_ as a nitrogen source and MgSO_4_ as an additional sulphate source was used to conduct the enrichment culture and biodegradation assays. The modified MSM composition per litre of deionized water is as follows: 500 mg KH_2_PO_4_, 500 mg K_2_HPO_4_, 1000 mg (NH_4_)_2_HPO_4_, 10 mg NaCl, 200 mg MgCl_2_·6H_2_O, 20 mg CaCl_2_, 0.339 mg MnSO_4_, 0.428 mg ZnSO_4_, 0.347 mg (NH_4_)_6_Mo_7_O_24_·4H_2_O), 0.4 mg CoCl_2_·6H_2_O and 15 mg EDTA with a pH of 7.2–7.4. After autoclaving at 121°C for 20 min 500 mg L^−1^ of sterile MgSO_4_ was added.

### Enrichment cultures and isolation of potential IBU‐degrading strains

Strains showing IBU‐degrading potential were obtained using enrichment cultures starting from environmental (soil, marine, and mine) samples and olive mill wastewater and its sediments. First, in a 250 ml Erlenmeyer flask, 6 g of each sample individually was mixed with 60 ml of MSM and 100 mg L^−1^ of IBU as the sole carbon and energy source, and the cultures were incubated with orbital shaking at room temperature. Then, a total of two successive enrichments were performed by re‐inoculating 10% (v/v) of the volume of the previous culture at 6 days (first enrichment) and 21 days (second enrichment). After the first and second enrichment, 100 μl of the culture was plated on MSM agar medium supplemented with 20 mg L^−1^ of IBU. To prevent bacterial growth in the solid medium due to impurities from the agar that could serve as carbon sources, we have used Ultrapure Noble Agar (Thermo Scientific, Ref. J10907, LOT: 213191). The grown bacterial colonies were then plated on LB medium to distinguish morphological differences until isolation was achieved. Isolated bacteria were labelled with codes according to the place (T: Tavira, ST: St Luzia, F: Fuseta, LO: Olive mill) and contaminant of isolation (IBU).

### Inoculum preparation for IBU removal/transformation experiments

The solubility of IBU in water is low (21 mg L^−1^ at 25°C), compared to the amount of carbon source generally used in bacterial growth media (e.g., 1000 mg L^−1^ glucose in LB medium). For this reason, the number of cells of the isolates was first multiplied in a culture medium before the biodegradation tests in solution. For bacterial growth, a colony of each isolate was incubated in sterile flasks with 100 ml of LB medium. The bacterial cultures were incubated overnight at 30°C on an orbital shaker at 160 rpm. Then, the bacterial cultures were centrifuged at 4000 rpm for 10 min. The pellet was washed with MSM to ensure that no traces of LB medium remained. Bacterial growth was monitored by the optical density (OD) at 600 nm at the beginning of the assay using a Hach Lange DR 2800 spectrophotometer.

### IBU removal/transformation experiments

These assays were carried out by inoculating 250 ml sterile Erlenmeyer flasks in triplicate with 50 ml of liquid MSM medium supplemented with IBU, as the sole carbon source, at an initial concentration of 5 mg L^−1^. Flasks without bacterial inoculation were used as a negative control to observe potential abiotic removal or transformation. The tests were inoculated with the amount of bacterial inoculum needed to reach a value of 1 for the OD at 600 nm (OD_600_). Then, they were agitated at 160 rpm and 30°C for 14 days. For analysis, at different times 2 ml aliquots were taken and centrifuged, and the supernatant was filtered using 0.22 μm PES sterile syringe filters (VWR, China). To ensure that the drug did not remain on the filter, 1 ml was discarded at the beginning. The filtered samples were stored at 4°C until HPLC analysis. The bioremoval percentage was obtained by considering the negative control and the residual concentration at each sampling time.

The concentration of IBU was quantified using an Ultra High Performance Liquid Chromatography Nexera system (SHIMADZU), equipped with an XBridgeTM C18 5 μm, 4.6 × 250 mm column (Waters, USA). The mobile phase composition was Methanol: Water (80:20 v/v), adjusted at pH ~3 with orthophosphoric acid 85%, using an isocratic method. The flow rate was set at 1.3 ml min^−1^, with an injection volume of 200 μl. The total run time was 6.5 min. For IBU, the detection wavelength (*ʎ*) was 204 nm and the retention time was approximately 4.89 min. The concentration of IBU in the samples was determined by calculating the slope of the calibration curves, which were prepared using five concentrations from 5 to 1 mg L^−1^ in volumetric flasks, using IBU (2‐(4‐Isobutylphenyl) propanoic acid, >98% purity) provided by Sigma‐Aldrich (Ref. 14883‐10G; LOT: SLCH8939).

Aiming to identify putative IBU metabolites generated in biodegradation experiments, samples were analysed by liquid chromatography–mass spectrometry (LC–MS) using a Thermo Scientific UHPLC, model Ultimate 3000, with a Thermo Scientific Accucore™ aQ C18 Polar Endcapped HPLC Column, with a column oven temperature of 25°C, a sampler temperature kept at 6°C and an injection volume of 10 μl. The mobile phase composition was water (A) and acetonitrile (B), both containing 0.1% of formic acid. The gradient (in v/v %) started with 100% of A for 2 min. Then, B increased linearly to 30% in 5 min (until minute 7), then B increased to 100% in 4 min (until minute 11) and B was maintained at 100% for an additional 4 min (until minute 15). Afterwards, the mobile phase returned to 100% of A in 1 min (until min 16), and then was maintained at 100% of A for 4 min (until min 20). The flow rate was 0.3 ml min^−1^. The absorbances were analysed using a Thermo Scientific Diode Array Detector, model Dionex UltiMate 3000 RS. Mass analysis was performed on a Thermo Scientific HRMS^n^ system, model Orbitrap Elite, equipped with electrospray ionization (ESI) and atmospheric pressure chemical ionization ion sources. Fragmentation spectra were using ESI obtained by running the system in data‐dependent mode using dynamic exclusion, in negative polarity. HR‐MS^n^ data was acquired using the following ionization parameters: capillary (ion transfer tube) temperature = 325°C; source heater temperature = 325°C; sheath gas flow = 35 arbitrary units; auxiliary gas flow = 5 arbitrary units; negative polarity with the source of voltage = 3.2 kV, source of current = 100 μA and S‐Lens RF level of 64.4%; scan mass range was 50–1000 *m*/*z*. LC–MS profiles and LC‐chromatograms were analysed using the Qual Browser application of the Thermo Scientific Xcalibur 4.1.50 software (Thermo Fisher Scientific Inc.). Then, possible compounds making the peak(s) emerging as the IBU concentration drops were proposed based on the precursor *m*/*z* and masses of IBU intermediate biodegradation compounds or possible conjugates.

### Studies on biodegradation kinetic models

The IBU removal/transformation curves were analysed and fitted to the best biodegradation kinetic model using an Excel spreadsheet developed by the FOCUS working group (FOCUS, 2006), which provides a guideline for calculating the degradation kinetic rates of environmental samples. The Solver tool and a statistical package from Microsoft were used for this purpose.

The biodegradation curves were fitted to a single first‐order (SFO) kinetics model using the parameters recommended by the FOCUS group and employing the least squares method.

$$\left(\mathrm{SFO}\right):M=M_{0}e^{-\mathrm{kt}};{\mathrm{DT}}_{50}=\mathrm{Ln}2/k;{\mathrm{DT}}_{90}\;\mathrm{Ln}10/k$$

In the equations, M and M_0_ represent the concentration of IBU (mg L^−1^) at time t and the initial concentration, respectively. The rate constant of degradation is denoted by k (day^−1^). The DT_50_ and DT_90_ reflect the time required by the bacteria to degrade 50 and 90% of the initial contaminating drug, respectively. The Chi‐square (*χ* ^2^) test is used as a tool to compare the goodness of fit of kinetic models by assessing the deviations between observed and calculated values. A suitable model should pass the significance level of 5% or less. The selection of these models was based on their relative simplicity, while still having the potential to accurately fit the measured dissipation kinetic datasets for both monophasic and biphasic biodegradation (Beulke et al., 2005).

### Taxonomic classification of bacterial strains showing IBU degrading potential

The best IBU‐removing bacterial strains were classified using 16S rRNA gene sequences. DNA extraction was carried out from each pure bacterial culture in LB liquid medium using the Nzytech microbial gDNA isolation kit (Nzytech, Portugal). The DNA concentration was quantified using an ultratrace UV spectrophotometer Nanodrop 1000 (Thermo Scientific). To amplify the 16S rRNA gene, PCR was performed using the Supreme NZYTaq 2x Green Master Mix (Nzytech, Portugal) and universal prokaryotic primers: Primer forward, 8F (5′‐AGA GTT TGATCC TGG CTC AG‐3′) (Weisburg et al., 1991) and Primer reverse, 1492R (5′‐GGT TAC CTT GTTACG ACT T‐3′) (Lane, 1991). The amplified PCR product was analysed by electrophoresis on a 1% agarose gel in 1x TAE buffer (AMRESCO, Solon, USA). Subsequently, the amplified PCR product was sequenced by the Sanger method (Sanger et al., 1977) in both directions using the Genetic Analyser Model 3130xl capillary electrophoresis sequencing system (Applied Biosystems, Foster City, USA). For sequence editing and assembly, the BioEdit 7.2 software (BioEdit Sequence Alignment Editor) was used (Alzohairy, 2011). The taxonomic classification was performed by comparing the sequences with the NCBI (National Center for Biotechnology Information) database using the Blastn program (Altschul et al., 1990).

### Bacterial growth curves and relation of OD_600_ to colony‐forming units

The pre‐inoculum of the selected best IBU‐removing bacteria, obtained from an overnight culture, was used to inoculate 100 ml of LB medium in a sterile 250 ml Erlenmeyer flask. Bacterial growth was monitored by measuring the OD_600_ every 2 h until 68 h with a Hach Lange DR 2800 spectrophotometer. The initial OD_600_ of the cultures was set to 0.1 and incubation was with agitation at 125 rpm and 30°C. To count the colony‐forming units (CFUs), serial dilutions were performed in LB medium from 10^−4^ to 10^−11^ and plated in solid LB medium. Two drops of 20 μl each from each dilution were plated on divided plates, as shown in Supplementary Material 1S. After 48 h, the number of CFU ml^−1^ was determined. The specific growth rate (μ) was calculated using the slope of a semilogarithmic curve during the exponential phase, and the duplication time (td) was calculated with the equation td = ln2/μ, where the natural logarithm of two is divided by the specific growth rate. The grow curves are a contribution to the strains' characterization and the estimated numbers of viable bacterial cells per OD values will be useful when preparing inocula in future assays.

### Genome sequencing and gene annotation of selected bacteria

The following steps were performed to sequence the genome of *K. pneumoniae* TIBU2.1, one of the selected best IBU‐removing/transforming bacteria. First, the bacterium was inoculated in 5 ml of LB liquid medium and incubated at 30°C at 150 rpm for 24 h. Then, the cells were harvested by centrifugation at 10,000 rpm for 3 min and their genomic DNA was isolated using NZY microbial gDNA isolation kit (Nzytech, Portugal). The DNA concentration was measured by an ultra‐trace UV spectrophotometer (Nanodrop 1000, Thermo Scientific). Next, the genome was sequenced by Integrated Microbiome Resource (Dalhousie University, Canada) using PacBio Sequel with a coverage range of 33×–203×. The sequencing reads were assembled by PacBio's SMRTlink genome assembler version 10.1 and annotated by the RAST tool (Aziz et al., 2018) and NCBI Prokaryotic Genome Annotation Pipeline.

### Identification of candidate IBU catabolic genes

tblastn was used to align the reference protein sequences related to the IBU degradation pathway obtained from literature and databases with the *K. pneumoniae* TIBU2.1 and *M. aubagnense* HPB1.1 genome. The reference proteins used were those described by Aulestia et al. (2022) in a work on the genetic characterization of the IBU degradative pathway of *Rhizorhabdus wittichii*. The default settings of the tblastn algorithm were applied (Max target sequences: 100, Expect threshold: 0.05, Word size: 5, Max matches in a query range: 0, Matrix: BLOSUM62, Gap costs: Existence: 11 Extension: 1, Compositional adjustments: conditional compositional score matrix adjustment, Filter: low complexity regions). Thus, the selection of potential catabolic genes was based on the high coverage and identity of the obtained tblastn hits. Moreover, in some cases with relatively lower identity, the catalytic domains of the chosen proteins were analysed using the Conserved Domain Database (CDD 2) and InterProScan (Jones et al., 2014).

## RESULTS

### Enrichment cultures and isolation of potential IBU degrading strains

A total of 11 colonies were isolated from selective plates inoculated with aliquots from the enriched cultures initially inoculated with estuarine sediments, soil samples exposed to aromatic hydrocarbons, and industrial olive mill wastewater and its sediments. The isolates were named based on the place of inoculum (Tavira [T], Sta. Luzia [ST], Fuseta [F], and Olive sludge [LO]), followed by the PC acronym (IBU), number of enrichment cultures (first [1] or second [2]), and successive numbers: TIBU2.1, TIBU2.2, TIBU2.3, TIBU2.4, TIBU2.5, STIBU2.1, STIBU2.2, STIBU2.3, FIBU2.1, LOIBU1.1, and LOIBU1.2. These colonies showed the ability to grow on MSM plates supplemented with IBU as the sole carbon source, which is an indication to consider them as possible IBU degraders.

### IBU removal/transformation experiments and studies on degradation kinetic models

**FIGURE 1:**
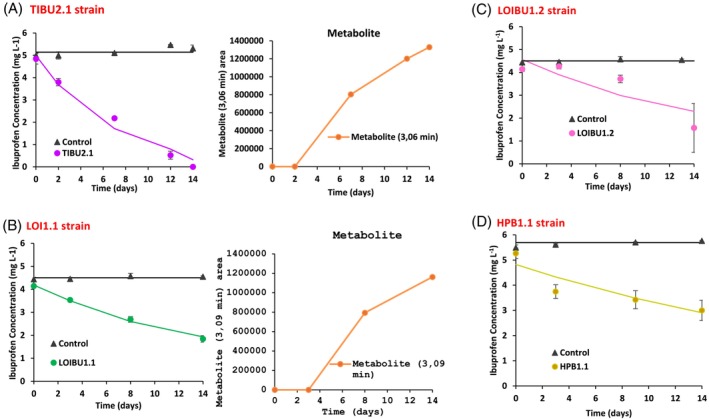
Biodegradation curves for IBU in solution in the presence of (A) TIBU2.1 strain and peak area of the metabolite detected at 3.06 min; (B) LOI1.1 strain and peak area of the metabolite detected at 3.09 min; (C) LOI1.2 strain and (D) HPB1.1 strain. The error bars represent the average deviation for three replicates. When error bars are not visible, it means they are smaller than the data point markers. Solid lines show the model fitting to the experimental results (symbols).

The 11 isolated strains mentioned above were used to carry out IBU removal/transformation tests in solution. However, only three showed a removal percentage higher than 15% at the end of the assay (14 days) and were chosen for degradation kinetic model studies: TIBU2.1, LOIBU1.1, and LOIBU1.2 (Figure 1). This relatively low number of selected strains shows the importance of the tests in liquid media. The reason for this was not investigated, but an obvious possibility is that some strains forming colonies on the selective solid medium may have used agar as a carbon source and not IBU. Moreover, in liquid media, the carbon source (drug) is more readily accessible to bacteria, whereas in agar plates, the bioavailability of the carbon source is limited (Bonnet et al., 2020). Therefore, in the liquid medium, the differences between bacteria regarding the efficiency in using the compound and/or the resistance to it become more evident. In what concerns the additional bacterial strain previously isolated through hydroquinone enrichment cultures, *M. aubagnense* HPB1.1, the results also reveal the good ability to remove IBU from the solution. The IBU concentration values and model curves obtained in tests with these four strains are shown in Figure 1A–D, while the corresponding model kinetic parameters are given in Table 1. In all assays, the IBU concentration in the negative control remained stable, indicating the absence of abiotic removal (binding to the flasks or chemical transformation).

**TABLE 1:** Kinetic parameters obtained from IBU biodegradation in solution after inoculation with IBU‐degrading bacterial strains.

Strain	*k* (day^−1^)	DT_50_ (days)	X^2^	Extent degraded (%)
TIBU2.1	0.153	5	12.1	100.0
LOI1.1	0.059	12	1.8	59.5
LOI1.2	0.053	13	13.9	65.5
HPB1.1	0.036	19	7.2	57.0

*Note*: Biodegradation curves were fitted to single first‐order model. K: mineralization rate constants. DT50: Time required for the concentration to decline to half of the initial value. X2 calculated values <X2 corresponding to the tabulated value (*p* < 0.05).

During the HPLC analysis to estimate IBU concentrations in samples from the biodegradation tests, attempts were also made to identify peaks that appeared as the IBU concentration decreased. This trend was only clear and consistent for one peak (retention time of 3.06–3.09 min) that became more prominent as the IBU concentration dropped. This peak appeared on the downwards slope of a larger peak appearing at ~2.5 min that was considered to be an injection peak since it was present in all samples, including IBU standards and just MSM mineral medium controls (as seen in Supplementary Material 3SA). To minimize the interference of this peak at ~2.5 min in the calculation of the area of the peak at 3.09 min, we manually made the baseline of the latter correspond to the descending slope of the former. For that, the tools for peak analysis of the native HPLC software were used, which allow automatic calculation of the peak area when the baseline is defined by the user.

In addition, samples were analysed by LC–MS for the identification of probable compound(s) making this IBU transformation product peak. Comparing the evolution of HPLC and LC–MS peaks along the incubation time allowed establishing the correspondence of the HPLC peak at 3.06–3.09 min with the LC–MS peak at 12.28–12.31 min. Then, considering the IBU mass (MW ~206) and considering the highest *m*/*z* of ~361 in the spectrum obtained for the IBU transformation product peak by LC–MS with negative ionization, the identification of compound(s) making the peak was focused on possible conjugates between known IBU biodegradation intermediates or conjugates between these and glucuronic acid (known to form conjugates with NSAIDs).

Five possibilities can be proposed for the appearing peak regarding conjugates between IBU biodegradation intermediates matching the precursor's putative mass (~362 g/mol) detected in the emerging peak:

- if 6‐carboxy‐5‐formyl‐6‐hydroxyhepta‐2,4‐dienoic acid (MW ~214), before further hydroxylation to form 6‐carboxy‐5‐formyl‐2,6‐dihydroxyhepta‐2,4‐dienoic acid, conjugates with 4‐Isobutylcatechol (MW ~166), losing a water molecule (MW ~18), a compound with MW = ~362 is formed;
- if 6‐carboxy‐5‐formyl‐6‐hydroxyhepta‐2,4‐dienoic acid (MW ~214), before further hydroxylation to form 6‐carboxy‐5‐formyl‐2,6‐dihydroxyhepta‐2,4‐dienoate, conjugates with 2‐(4‐Hydroxyphenyl)propanoic acid (MW ~166), losing a water molecule (MW ~18), a compound with MW = ~362 is formed;
- if 5‐Formyl‐2‐hydroxy‐7‐methylocta‐2,4‐dienoic acid (MW ~198) conjugates with 5‐Formyl‐7‐methylocta‐2,4‐dienoic acid (MW ~182), losing a water molecule (MW ~18), a compound with MW = ~362 is formed;
- if Isobutylcatechol with an additional hydroxyl group (MW ~182) conjugates with 5‐formyl‐2‐hydroxy‐7‐methylocta‐2,4‐dienoic acid (MW ~198), losing a water molecule (MW ~18), a compound with MW = ~362 is formed;
- if 2‐(4‐Hydroxyphenyl)propanoic acid with an additional hydroxyl group (MW ~182) conjugates with 5‐formyl‐2‐hydroxy‐7‐methylocta‐2,4‐dienoic acid (MW ~198), losing a water molecule (MW ~18), a compound with MW = ~362 is formed.

On the other hand, NSAIDs can be eliminated in animals through conjugation with polar sugar moieties to form glucuronides (Kuehl et al., 2005). Indeed, it is known that IBU is metabolized by oxidation to carboxyibuprofen and hydroxyibuprofen and by conjugation to an acyl glucuronide, forming IBU acyl glucuronide (Castillo et al., 1995). Besides, many bacteria contain glucuronic acid as a major component of extracellular polysaccharides (Zhao et al., 2016). Therefore, as a possible hypothesis, it can be suggested that the strains in the tests in which the IBU transformation product peak produce glucuronic acid that combines with IBU biodegradation intermediates. In this case, the conjugation with intermediates is proposed instead of conjugation with IBU itself, because the mass of IBU acyl glucuronide conjugate (MWt ~382) is much larger than the largest ion obtained in the LC–MS ionization for the detected emerging peak (*m*/*z* ~ 361). Given this, two additional possible compounds can be proposed for that peak:

vi if Isobutylcatechol with an additional hydroxyl group (MW ~182) conjugates with glucuronic acid (MWt ~194), losing a water molecule (MW ~18), a compound with MW = ~358 is formed;
vii if 2‐(4‐Hydroxyphenyl)propanoic acid with an additional hydroxyl group (MWt ~182) conjugates with glucuronic acid (MW ~194), losing a water molecule (MW ~18), a compound with MW = ~358 is formed.

Nevertheless, compared to those proposed above (i–v), these two compounds (vi and vii) seem less probable since there is a difference of 4 g/mol between their masses to the precursor's putative mass (~362 g/mol for an *m*/*z* of ~361 with negative ionization). When just the masses (minus 1) of the proposed conjugates i–v (~361) and conjugates vi and vii (~357) are displayed independently in the chromatograms of samples along the test with the TIBU2.1 strain, it is possible to see that in the chromatograms displaying *m*/*z* = ~361 the intensity signal increases at the retention time of the peak detected as IBU transformation product (the peak at 12.3 min had an intensity around 300 at day 0 and reached an intensity over 3000 at day 12), while in the chromatograms displaying *m*/*z* = ~358, four peaks are appearing at different retention times and with low‐intensity signal. This shows even more clearly that the proposed IBU transformation products i–v are more likely to be the ones detected compared to vi and vii. On the other hand, when just the masses (minus 1) of the compounds making the proposed conjugates are independently displayed, it is possible to see that in the chromatograms with *m*/*z* values of ~165, ~181, and ~197 peaks are growing along the incubation time, while in the chromatograms with *m*/*z* values ~193, ~213, and ~229 there are not evident growing peaks. These results point to the IBU transformation products detected being conjugates i, ii, iii, iv, or v; but in addition, they point to a greater probability of being the conjugates iii, iv, or v, compared to i and ii.

**FIGURE 2:**
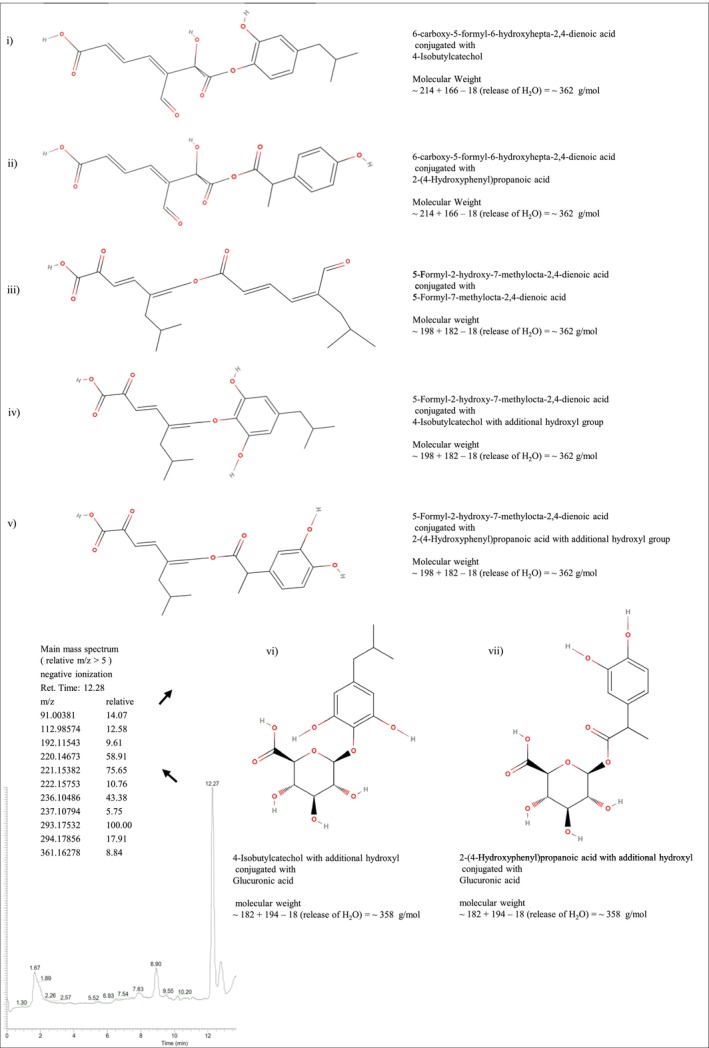
Chromatogram obtained by LC–MS analysis with a sample from the IBU biodegradation test with TIBU2.1 strain collected on incubation day 14, with the emerging peak of IBU transformation product(s) marked with an arrow to the respective mass spectrum of most abundant ions (relative >5) and to possible compounds making the peak—with masses close to the largest ion *m*/*z* (~361).

Figure 2 shows a summarized mass spectrum just with the most abundant ions (relative intensity >5) of the detected emerging peak and the conjugates proposed as possible IBU transformation product(s) making the peak. Supplementary Material 2S shows the mass spectrum obtained in the emerging peak and the proposed ionization steps for the three main ions; the structural formulas of IBU, gucuronic acid, IBU acyl glucuronide, and the suggested intermediates that can combine, or combine with glucuronic acid, to form conjugates with a molecular weight close to 361; as well as chromatograms of samples along the test with the TIBU2.1 strain, displaying the masses (minus 1) of the compounds making the proposed conjugates and of those conjugates.

TIBU2.1 presented the best IBU concentration drop (Figure 1A, Table 1), with a complete removal achieved after 14 days, and a DT_50_ value of only 5 days. The metabolite(s) peak detected in the presence of the TIBU2.1 at 3.06 min, as shown in Figure 1A, was observed after 7 days of incubation when 57% of IBU had already been degraded. The area of this peak began to increase as the parent compound (IBU) was degraded until day 14 when 100% of IBU had been degraded (Figure 1A). This growing trend until the end of the experiment suggests the studied bacterium could not degrade the corresponding compound(s).

LOIBU1.1 and LOIBU1.2 were able to remove 59.4 ± 0.1% (Figure 1B) and 65.5 ± 1.1% (Figure 1C) of the initial IBU concentration, respectively. Plus, compared to the TIBU2.1 strain, a longer time was necessary to reach 50% removal (DT_50_ = 13 and 12 days, for LOIBU1.2 and LOIBU1.1, respectively). The metabolite(s) peak was detected in the test with LOIBU1.1 strain, with a retention time (3.09 min) that closely resembles that found with TIBU2.1 (Figure 1B). This suggests that it could be caused by the same compound(s). The peak was identified for the first time after 3 days, when 14.5% of IBU was removed, and then followed a growing trend until the end of the experiment (Figure 1B), as observed with the TIBU2.1 strain. In the case of LOIBU1.2, the metabolite(s) peak was not detected (Figure 1C).

Finally, up to 57 ± 0.4% of the IBU was removed by *M. aubagnense* HPB1.1 after 14 days (Figure 1D), with a DT_50_ value of 19 days (Table 1), and the metabolite's peak was also not detected in this case.

### Bacterial growth curves and relation of OD_600_ to CFUs

Supplementary Material 4S illustrates the growth curves for the four bacteria studied, revealing the absence of a Lag phase in all of them. In addition, the graphs report insights into the CFU per millilitre based on OD. Table 2 shows the growth rates (μ) and duplication times (td), calculated during the exponential phase for the 4 IBU‐removing bacteria. The bacterium TIBU2.1 presented the highest growth rate μ of 0.6 h^−1^, corresponding to a td of 1.2 h. The LOIBU1.1 and LOIBU1.2 strains showed intermediate growth rates between 0.4 and 0.6 h^−1^ with duplication times from 1.2 to 1.7 h. On the other hand, *M. aubagnense* HPB1.1 showed the lowest μ of 0.1 h^−1^ and a td of 5.9 h. These findings highlight differences in the growth kinetics among the isolated bacteria, and this information will be useful estimate the number of bacterial cells that correspond to a specific OD when preparing inocula in future assays.

**TABLE 2:** Kinetics growth rate and duplication time of isolated degrading bacteria.

Isolated bacteria	TIBU2.1	LOIBU1.1	LOIBU1.2	*M. aubagnense* HPB1.1
Growth rate μ (h^−1^)	0.6	0.6	0.4	0.1
Duplication time td (h)	1.2	1.2	1.7	5.9

### Taxonomic classification of bacterial strains showing IBU degrading potential

After the IBU removal/transformation tests, it was decided to identify only those bacteria that showed a high removal of the study drug. The 16S rRNA gene amplicons of TIBU2.1, LOIBU1.1, and LOIBU1.2 showed a match over the minimum requirement of 98.7% (Yarza et al., 2014) to bacteria species in the NCBI GenBank database (Table 3). The results showed that all of them belong to the phylum Pseudomonadota and the class Gammaproteobacteria. Two of the isolates belong to the *Klebsiella* genus, while the other one belongs to the *Pseudomonas* genus. Specifically, TIBU2.1 exhibited a 99.71% identity match with *K. pneumoniae*, LOIBU1.1 showed an identity of 99.46% with *Klebsiella variicola*, and LOIBU1.2 corresponded to *Pseudomonas aeruginosa* with an identity match of 99.86%.

**TABLE 3:** Phylogenetic affiliations of ibuprofen‐degrading newly isolated bacteria, based on 16S gene sequence alignments using the NCBI's blastn application on the rRNA/ITS database 16S ribosomal RNA sequences (Bacteria and Archaea).

Strain	16S sequence (GenBank accession)	Similarity (%)	Phylum/class, family, genus, species
TIBU2.1	PP437704	99.71	Pseudomonadota/Gammaproteobacteria, Enterobacteriaceae, *Klebsiella*, *pneumoniae*
LOIBU1.1	PP446156	99.46	Pseudomonadota/Gammaproteobacteria, Enterobacteriaceae, *Klebsiella*, *variicola*
LOIBU1.2	PP438318	99.86	Pseudomonadota/Gammaproteobacteria, Pseudomonadaceae, *Pseudomonas*, *aeruginosa*

### Genome sequencing and gene annotation of selected bacteria

The two bacterial strains *K. pneumoniae* TIBU2.1 and *M. aubagnense* HPB1.1 were selected for in silico genomic studies due to their promising results on the IBU removal tests in solution and novelty regarding species reported as able to biodegrade IBU. Such studies aimed to identify candidate genes involved in the IBU degradation pathway.

The whole genome sequencing data of *K. pneumoniae* TIBU2 was submitted to the NCBI database under BioPorject PRJNA1049394 and GenBank accession numbers CP139935.1 and CP139936.1. The assembled genome consists of two contigs, one of them corresponding to a circular chromosome with a total size of 5,188,999 bp (CP139935.1), and the other corresponding to a plasmid (167,501 bp). It has a guanine‐cytosine (GC) content of 57.4% and 4973 coding genes are present in the draft genome, including 9 5s rRNAs, 8 16s rRNAs, 8 23s rRNAs, and 89 tRNAs.

The genome of the other strain selected for in silico genomic studies, *M. aubagnense* HPB1.1, was already sequenced in a previous study on Paracetamol and Hydroquinone biodegradation and deposited in the NCBI (BioProject PRJNA955654 and GenBank accession numbers CP122994 to CP122999). The assembled genome has a total size of 6,224,392 bp. It comprises six contigs: a circular chromosome (CP122994) measuring 5,734,470 bp, three circular plasmids (CP122995, CP122998, CP122999) of 168,657 bp; 23,691 bp; and 16,144 bp, and two linear sequences, likely unclosed assemblies of circular plasmids (CP122996, CP122997), with lengths of 181,565 bp and 99,865 bp, respectively. The GC content of the HPB1.1 genome is 66.4%, and it has been annotated with 5838 genes.

Both these strains have been made available for the global research community in the open collection of the Agricultural Research Service Culture Collection (NRRL) in the United States (https://nrrl.ncaur.usda.gov/). The NRRL accession assigned to *M. aubagnense* HPB1.1 is B‐65706 and the NRRL accession assigned to *K. pneumoniae* TIBU2.1 is B‐65705.

**FIGURE 3:**
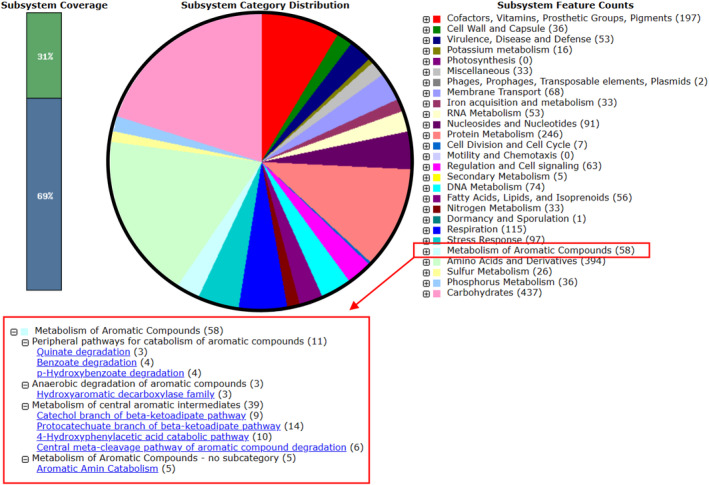
Subsystem category distribution of major protein‐coding genes of *K. pneumoniae* TIBU2.1 as annotated by the RAST annotation server. The bar chart shows the subsystem coverage in percentage (the blue bar corresponds to the percentage of proteins included). The pie chart shows the percentage distribution of the 27 most abundant subsystem categories.

**FIGURE 4:**
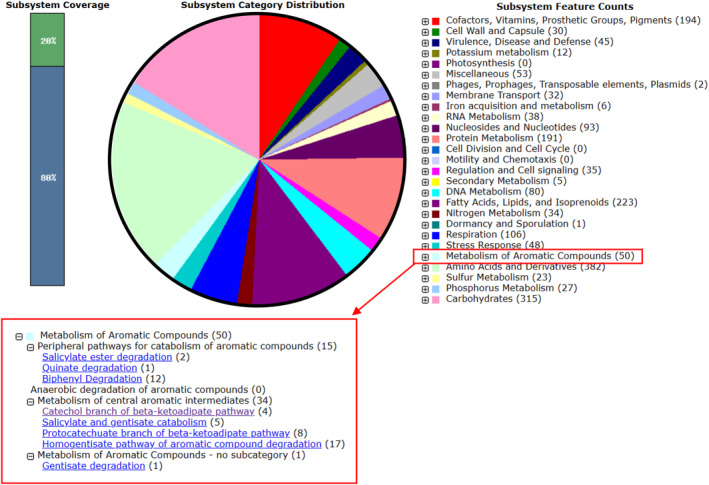
Subsystem category distribution of major protein‐coding genes of *M. aubagnense* HPB1.1 as annotated by the RAST annotation server. The bar chart shows the subsystem coverage in percentage (the blue bar corresponds to the percentage of proteins included). The pie chart shows the percentage distribution of the 20 most abundant subsystem categories.

The metabolism of aromatic compounds was seen more in detail for a general depiction of both these strains' genetic potential to catabolize persistent organics. A total of 58 genes involved in the metabolism of aromatic compounds were identified in the genome of TIBU2.1 (Figure 3) and 50 in HPB1.1 (Figure 4). These genes are classified into three subcategories: (1) peripheral pathways for catabolism of aromatic compounds, (2) anaerobic degradation of aromatic compounds, and (3) metabolism of central aromatic intermediates. The peripheral category (1) genes are related to the degradation of quinate, benzoate, p‐hydroxybenzoate, salicylate ester, and biphenyl. The bacterium TIBU2.1 has 11 genes and HPB1.1 contains 15 genes in this category. On the other hand, in the anaerobic category (2), only the bacterium TIBU2.1 has three genes belonging to the hydroxyaromatic decarboxylase family. In the last category, in the central intermediates category (3), TIBU2.1 and HPB1.1 have 42 and 35 genes, respectively. These genes are involved in central metabolism, such as the catechol and protocatechuate branch of the beta‐ketoadipate pathway, central meta‐cleavage and homogentisate pathway, 4‐hydroxyphenylacetic acid catabolic pathway, and salicylate and gentisate catabolism.

### Identification of candidate IBU catabolic genes

To identify enzymes potentially involved in the IBU biodegradation pathways of *K. pneumoniae* TIBU2.1 and *M. aubagnense* HPB1.1, in silico searches were carried out to identify genetic homologies between proteins encoded in their genomes and proteins reported in the IBU degradation pathway suggested by Aulestia et al. (2022). The results, based on the percentage of similarity, were obtained by aligning the reference proteins individually with the genome of each bacterium using the tblastn application program on the NCBI web page and are shown in Table 4.

**TABLE 4:** Information about the alignment of proteins known to have a role in IBU biodegradation, described by Aulestia et al. (2022), used as query references, with the identified similar proteins encoded in the target genomes of *K. pneumoniae* TIBU2.1 and *M. aubagnense* HPB1.1.

Reference protein data				Alignment data				Annotate protein data	
Gene name	Protein (accession number)	Function NCBI	Bacteria strain	Coverage (%)	Identity (%)	E‐value	Peg annotation	Function annotation	
*ipfF*	WP_208634566.1	AMP‐binding protein	TIBU2.1	98	25	7e^−39^	3068	Long‐chain‐fatty‐acid—CoA ligase	
			HPB1.1	99	32	2e^−56^	1069	Long‐chain‐fatty‐acid—CoA ligase	
*ipfA*	WP_208634570.1	Aromatic ring‐hydroxylating dioxygenase subunit alpha	TIBU2.1	89	30	1e^−46^	2594	Benzoate 1,2‐dioxygenase alpha subunit	
			HPB1.1	98	45	1e^−111^	700	Putative dioxygenase hydroxylase component	
*ipfB*	WP_208634569.1	Aromatic‐ring‐hydroxylating dioxygenase subunit beta	TIBU2.1	66	31	6e^−09^	2593	Benzoate 1,2‐dioxygenase beta subunit	
			HPB1.1	90	44	6e^−36^	701	Benzoate 1,2‐dioxygenase beta subunit	
*ipfH*	WP_226870539.1	FAD‐dependent oxidoreductase	TIBU2.1	63	28	5e^−09^	2962	Nitrite reductase [NAD(P)H] large subunit	
			HPB1.1	91	32	5e^−47^	2700	Anthranilate dioxygenase reductase	
*ipfI*	WP_185210326.1	Non‐heme iron oxygenase ferredoxin	TIBU2.1	69	29	2e^−05^	2360	Vanillate O‐demethylase oxygenase subunit	
			HPB1.1	90	34	2e^−08^	2356	Pyruvate dehydrogenase	
*ipfD*	WP_226949243.1	Hypothetical protein	TIBU2.1	‐	‐	‐	‐	‐	
			HPB1.1	72	36	5e^−32^	760	Sterol carrier protein IgrF	
*ipfE*	WP_208634567.1	OB‐fold domain‐containing protein	TIBU2.1	‐	‐	‐	‐	‐	
			HPB1.1	51	38	4e^−08^	758	Acyl‐CoA transferase domain of IgrD/Nucleic‐acid‐binding domain of IgrD	
*ipfL*	WP_208634508.1	Catechol 2,3‐dioxygenase	TIBU2.1	‐	‐	‐	‐	‐	
			HPB1.1	‐	‐	‐	‐	‐	
*ipfM*	WP_208634595.1	2‐Hydroxymuconic semialdehyde dehydrogenase	TIBU2.1	100	39	1e^−106^	643	5‐carboxymethyl‐2‐hydroxymuconate semialdehyde dehydrogenase	
			HPB1.1	100	36	2e^−86^	4970	Aldehyde dehydrogenase	
*ipfP*	WP_208634520.1	Tautomerase family protein	TIBU2.1	‐	‐	‐	‐	‐	
			HPB1.1	‐	‐	‐	‐	‐	
*ipfO*	WP_208634521.1	2‐oxo‐3‐hexenedioate decarboxylase	TIBU2.1	97	36	2e^−43^	640	2‐oxo‐hepta‐3‐ene‐1,7‐dioic acid hydratase	
			HPB1.1	97	36	2e^−37^	4266	2‐oxopent‐4‐dienoate hydratase	
*ipfN*	WP_208634522.1	Fumarylacetoacetate hydrolase family protein	TIBU2.1	98	40	4e^−56^	2852	2‐keto‐4‐pentenoate hydratase	
			HPB1.1	97	43	4e^−51^	4266	2‐oxopent‐4‐dienoate hydratase	
*ipfS*	WP_208634518.1	4‐Hydroxy‐2‐oxovalerate aldolase	TIBU2.1	95	48	1e^−102^	2850	4‐hydroxy‐2‐oxovalerate aldolase	
			HPB1.1	95	48	2e^−94^	4268	4‐hydroxy‐2‐oxovalerate aldolase	
*ipfQ*	WP_208634519.1	Acetaldehyde dehydrogenase (acetylating)	TIBU2.1	94	55	7e^−86^	2851	Acetaldehyde dehydrogenase, acetylating, in gene cluster for degradation of phenols, cresols, catechol	
			HPB1.1	95	56	2e^−98^	768	Acetaldehyde dehydrogenase, acetylating, (EC 1.2.1.10) in gene cluster for degradation of phenols, cresols, catechol	
*ipfT*	WP_208634517.1	Acyl‐CoA dehydrogenase	TIBU2.1	49	28	5e^−18^	424	Alkylation response protein AidB, acyl‐CoA dehydrogenase family	
			HPB1.1	87	37	2e^−71^	2157	3‐methylmercaptopropionyl‐CoA dehydrogenase (DmdC)	

*Note*: (‐) Not detected.

The percentage of identity between homologous proteins from different bacterial species can vary depending on the evolutionary distance. Though there is no precise threshold for the percentage of identity needed to infer functional similarity among homologous proteins from different bacteria, research suggests that identity percentages above 40% indicate high conservation of biochemical function among homologues, while below 30% suggest significant functional variation (Todd et al., 2001). Thus, for sequences with lower identity percentages indicating significant evolutionary divergence, studying protein structures is crucial for comprehending the functional differences (Pearson, 2014). According to Galperin and Koonin (2012), enzymes within a superfamily generally exhibit common sequence motifs, corresponding to crucial active site residues, and frequently possess predicted reaction mechanisms. Consequently, an examination of the catalytic domains has been conducted for the identified proteins (tblastn hits) with alignment identity percentages below 40%.

Enzymes such as IBU‐CoA ligase, aliphatic monooxygenases, hydroxylation enzymes, and dioxygenases have been associated with the IBU metabolic pathway (Aulestia et al., 2022; Marchlewicz et al., 2017; Murdoch & Hay, 2005). The first step is catalysed by the enzyme ibuprofenyl‐CoA ligase (encoded by gene *ipfF*), an AMP‐binding protein that catalyses the attachment of IBU to coenzyme A. The AMP‐binding protein (Accession number: WP_208634566.1) described for *Sphingomonadaceae* was compared with the genome sequences of TIBU2.1 and HPB1.1. The percentage of identity between the query and the subject hits was 25 and 32% with coverages of 98 and 99%, respectively (Table 4). Interestingly, both the hits share the same functional annotation: Long‐chain‐fatty‐acid‐CoA ligase. Additionally, the specific residues involved in the active sites were examined in the alignments (Supplementary Material 5S). First, the catalytic domain of the protein was verified using the CDD (Supplementary Material 5SB), and then the corresponding active sites were identified on the candidate AMP‐binding proteins of each studied strain (Supplementary Material 5S, A1 and A2). The alignments exhibit conserved amino acids (in red bold) in the active sites associated with the investigated function in both strains, suggesting that both encoded proteins can be considered candidates for enzymes that initiate the IBU biodegradation process, despite the evolutionary differences with the enzyme described for *Sphingomonadaceae* and used as reference (<40% identity).

In the second step of the IBU degradation pathway described by Aulestia et al. (2022), IBU bound to coenzyme A is attacked by a dioxygenase system encoded by the *ipfABHI* genes, to form the dihydrodiol. This group of proteins was individually used for searches in the studied genomes, namely: the aromatic ring‐hydroxylating dioxygenase α subunit (IpfA, Accession number: WP_208634570.1), the aromatic‐ring‐hydroxylating dioxygenase β subunit (IpfB, Accession number: WP_208634569.1), the FAD‐dependent oxidoreductase (IpfH, Accession number: WP_226870539.1), and non‐heme iron oxygenase ferredoxin (IpfI, Accession number: WP_185210326.1). The TIBU2.1 genome revealed hits for the entire group of proteins, showing identity percentages of 30, 31, 28, and 29%, for *ipfA*, *B*, *H*, and I, respectively. Despite their identity percentages being below 40% when compared to query reference proteins, the functional annotation reveals them to be a dioxygenase α subunit, a dioxygenase β subunit, a reductase subunit, and an oxygenase subunit, respectively. Moreover, the alignment between the reference IpfA protein and respective hit in the TIBU2.1 genome reveals that the amino acid residues involved in the active sites are conserved (Supplementary Material 6S, A1), which is another indication that this protein may be a candidate for the dioxygenase α subunit in the IBU degradation pathway of this strain. For the hit found as a putative β subunit (IpfB) encoded protein in TIBU2.1, the identity percentage of 31% (much less than 40%) also prompts a study of the protein's active sites. However, the active site of the protein aromatic‐ring‐hydroxylating dioxygenase β subunit is not well‐defined. Indeed, the active site of these enzymes is typically in the α subunit, which contains a non‐heme ferrous ion coordinated by three ligands. The alpha subunit also has a β‐sheet domain that contains a Rieske [2Fe‐2S] centre, and the active‐site iron centre of one of the α subunits is directly connected by hydrogen bonds through a single amino acid to the Rieske [2Fe–2S] centre (Kauppi et al., 1998). Nevertheless, an important aspect to highlight is the position in the genome of genes encoding these proteins, arranged one after the other (PEG annotations positions 2594 and 2593; Table 3), which is typical for two subunits of an enzyme. In what concerns the hit for the FAD‐dependent oxidoreductase (IpfH) found in TIBU2.1, it also exhibits an identity percentage with the query (28%) that is far below the established limit of 40%. Plus, its active sites could not be studied because the specific amino acids involved in the active site of FAD‐dependent oxidoreductases can vary among different enzymes within this family, and there are no established active sites in the literature for the IpfH protein. Therefore, the gene hit identified in the TIBU2.1 strain for IpfH cannot be considered a candidate for FAD‐dependent oxidoreductase. In the case of the IpfI protein (non‐heme iron oxygenase ferredoxin), the active sites have been investigated, and it was observed that they remain conserved in hit found in the TIBU2.1 strain (Supplementary Material 7SA), thus maintaining the hypothesis of a similar function despite the 29% identity to the query reference protein. On the other hand, the bacterium HPB1.1 also exhibited similarity hits with these entire groups of query proteins (identity percentages 45, 44, 32, and 34%, respectively, for IpfA, IpfB, IpfH, and IpfI). The hits with high similarities for IpfA and IpfB suggest the presence of the two dioxygenases α and β subunits. These proteins correspond to those annotated as putative dioxygenase hydroxylase component and Benzoate 1,2‐dioxygenase β subunit, both arranged continuously (PEG annotation 700 and 701; Table 3) thus reinforcing the idea they are subunits of the same enzyme. Regarding the hit for IpfH in the HPB1.1 strain, again, the active sites of the identified protein could not be studied for the reason described earlier. Thus, the gene identified for this protein in the HPB1.1 strain cannot be considered an FAD‐dependent oxidoreductase candidate to be involved in IBU degradation. Nevertheless, the amino acids responsible for the active sites of the hit found for the IpfI protein were studied and confirmed to be conserved, thus suggesting a similar function (Supplementary Material 7S, A2).

Afterwards, IBU biodegradation involves the formation of the main central intermediates, isobutyl catechol, and propionyl‐CoA, through the action of the thiolase enzyme encoded by the *ipfDE* genes (Aguilar‐Romero et al., 2021; Aulestia et al., 2021). Thus, the presence of the proteins encoded by the *ipfDE* genes in the TIBU2.1 and HPB1.1 genomes was also analysed. The alignment of units encoded by *ipfD* and *ipfE* genes revealed hits for encoded proteins in the HPB1.1 strain with 36 and 38% identity, respectively; while no similarities were found in the TIBU2.1 strain. The specific amino acids involved in the active site of these proteins were not compared since they are not explicitly mentioned in the literature. Still, based on the criteria established by Todd et al. (2001), their function may be maintained since the sequence identities with the query reference protein are close to 40%. Thus, these hits can still be considered candidates for future molecular studies to determine whether they encode for the IpfDE proteins involved in IBU degradation.

The next studied step in IBU degradation is the opening of the aromatic ring of isobutyl catechol, catalysed by the enzyme catechol 2,3‐dioxygenase, encoded by the *ipfL* gene. However, no similarities were found between the IpfL protein and proteins encoded in the studied genomes. This could indicate that both the strains under study might have a different enzyme with a similar function to catalyse this step or follow alternative steps in the IBU‐degrading pathway, or they can transform IBU into intermediate products but not completely biodegrade this PC.

The following step depends on the activity of a semialdehyde dehydrogenase, encoded by the *ipfM* gene, in forming 2‐hydroxy‐5‐isobutylhexa‐2,4‐dienoic acid. TIBU2.1 has an encoded protein with 39% identity with the reported enzyme, while the HPB1.1 bacterium has a protein with 36% identity. Upon aligning the proteins encoded in the TIBU2.1 and HPB1.1 genomes with the reference semialdehyde dehydrogenase, it can be observed that the residues of the active sites are conserved (Supplementary Material 8S, A1 and A2), thus suggesting the possibility of similar function and putative involvement in the IBU degradation pathway.

Finally, once the candidate genes involved in the initial steps of degradation had been analysed, the study continued with enzymes associated with the final steps of IBU degradation, which involves metabolizing 2‐hydroxy‐5‐isobutylhexa‐2,4‐dienoic acid into end products that are readily metabolized by the tricarboxylic acid cycle and beta‐oxidation. The enzymes studied in this final step have activities of tautomerase, decarboxylase, hydratase, aldolase, aldehyde dehydrogenase (acylating), and acyl‐CoA dehydrogenase, and are encoded by *ipfP*, *ipfO*, *ipfN*, *ipfS*, *ipfQ*, and *ipfT* genes, respectively.

No similarities were found with the reference tautomerase enzyme (*ipfP* gene) and proteins encoded in the studied bacteria. However, similarities were detected between the reference enzymes encoded by the *ipfO*, *N*, *S*, *Q*, and *T* genes and proteins encoded by the TIBU2.1 strain (with 36, 40, 48, 55, and 28% identity, respectively) and by the HPB1.1 strain (with 36, 43, 48, 48, 56, and 37% identity, respectively). In most of them, the percentage of identity concerning the reference protein is above 35% in both bacterial strains, suggesting the presence of the genes that may encode functional enzymes for the transformation of IBU intermediate metabolites into small metabolic products that are less toxic and easier to degrade (Murdoch & Hay, 2013; Salgado et al., 2020). The exception is in the case of the IpfT protein in the TIBU2.1 strain, with 28% identity. Therefore, a study of the active sites was conducted for this protein, where it was observed that the amino acids constituting the active sites are conserved in different bacterial strains, including in the study strain TIBU2.1 (Supplementary Material 9S). This indicates that the found protein may have the same function as the reference protein, and thus can be considered a candidate for future functional studies.

**FIGURE 5:**
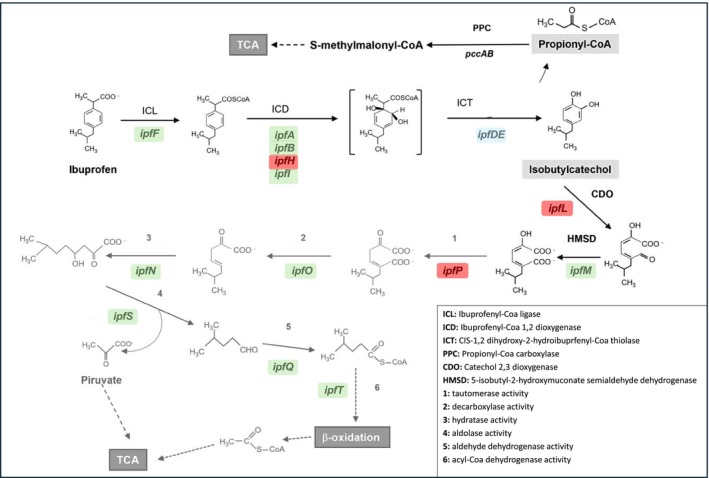
The metabolic pathway for IBU biodegradation proposed for *R. wittichii* MPO218, with the genes responsible for each stage, described by Aulestia et al. (2022). Genes highlighted in green correspond to those for which putative homologues (candidate catabolic genes for future functional studies) were identified in *K. pneumoniae* TIBU2.1 and *M. aubagnense HPB1*.1; highlighted in blue correspond to those identified in *M. aubagnense* HPB1.1 but not found in TIBU2.1; highlighted in red are those not found in both strains. TCA—tricarboxylic acid cycle.

Figure 5 summarizes the IBU metabolic route studied in the two bacterial strains selected in this work, highlighting the genes present in each of them that can be considered candidate genes for future functional studies.

## DISCUSSION

Three bacterial strains (*K. pneumoniae* TIBU2.1, *K. variicola* LOIBU1.1 and *P. aeruginosa* LOIBU1.2) isolated from enrichment cultures inoculated with environmental samples potentially exposed to hydrocarbons have shown potential to be IBU‐degrading bacteria, confirming that this type of sites may be a good source of microorganisms with interesting characteristics for biotechnological applications. Indeed, it has been demonstrated that matrices exposed to pollutants with aromatic rings for a long period can be useful for obtaining degrading microorganisms. The microbiota inhabiting those habitats and adapted to the presence of aromatic compounds can have some specimens with metabolic tools necessary to break and use persistent organic pollutants as a source of energy and carbon, having potential applications in bioremediation (Parajuli et al., 2017). Moreover, several studies have shown that high concentrations of such pollutants in enrichment cultures can promote the selective growth of degrading strains (Jia et al., 2020; Lara‐Moreno et al., 2022; Pápai et al., 2023).

In addition, the strain *M. aubagnense* HPB1.1 isolated from the wall moonmilk of a mine in a previous study also revealed good IBU‐removing capacity, confirming the generally accepted idea that there is a great biotechnological potential in microbial communities from extreme environments. Indeed, moonmilk, a white mud‐like exudate found in walls of some caves, is an extreme environment primarily composed of fine CaCO_3_ crystals where extremophiles and non‐extremophiles are found and from where strains with biotechnological potential have already been isolated (Hui et al., 2021; Kosznik‐Kwaśnicka et al., 2022).

It is worth emphasizing that this is the first time that the genera *Klebsiella* and *Mycolicibacterium* are reported as showing evidence of IBU‐degrading capacity. Nevertheless, the *Klebsiella* genus has been described in the literature by its potential to degrade toxic compounds, for instance: *Klebsiella oxytoca* NBA‐1, which was isolated from a PC WWTP, has demonstrated its ability to degrade toxic compounds such as nitrobenzene (Ren et al., 2015) and *Klebsiella* sp. KSC, a bacterium isolated from contaminated soil, was reported as a diclofenac‐degrading bacterium (70 mg L^−1^ in less than 72 h) (Stylianou et al., 2018). In another study, *Klebsiella* sp. CLX‐3, isolated from activated sludge, was able to eliminate over 99% of cefalexin at an initial concentration of 10 mg L^−1^ within 12 h (Tian et al., 2023). In particular, strains of this species, *K. pneumoniae*, have been described in the literature as able to degrade PCs (other than IBU). Moreover, several authors have reported *Klebsiella* spp. as tetracycline (TC) and chlortetracycline (CTC) degrading bacteria. For example, *K. pneumoniae* strain TR5, obtained from chicken manure through enrichment culture, demonstrated rapid degradation of TC, reaching approximately 90% degradation within 36 h, starting with an initial TC concentration of 200 mg L^−1^ (Yin et al., 2020). In another study, Al‐Dhabi and Arasu, (Al‐Dhabi & Arasu, 2022) reported that *K. pneumoniae* CH3, isolated from wastewater, achieved 99.4 ± 2.3% biodegradation of CTC from an initial concentration of 200 mg L^−1^. Also, a strain, isolated from pig manure, removed 95% of TC from synthetic wastewater (from 100 mg L^−1^ of initial concentration) (Jingrui et al., 2022). Moreover, *K. pneumoniae* WAH1 was described for its ability to remove diclofenac sodium; it achieved a degradation of 79% in 72 h from an initial concentration of 10 mg L^−1^ (Sharma et al., 2021). Interestingly, one of the other IBU‐removing bacterial strains (LOIBU1.1) isolated in this work was classified as *K. variicola*. This species shares a high degree of genetic similarity with *K. pneumoniae* (Rosenblueth et al., 2004) and it has also been recognized for its potential in biodegradation and bioremediation. For example, *K. variicola* has been used in the removal of phenols (Mahgoub et al., 2023), heavy metals (Das & Osborne, 2017), herbicides (Zhang et al., 2019), and dyes (Eslami et al., 2019). Yet, to date, no scientific articles have described the capacity of *K. variicola* to degrade PCs. In general, all these studies highlight the broad potential of *K. pneumoniae* and its closely related *K. variicola* in biotreatment and/or bioremediation efforts, showing diverse possible applications in waste treatment and environmental protection. In particular, the work presented here shows for the first time the potential of these two species in applications involving IBU biotreatment. Indeed, the detection of putative conjugates between IBU intermediate metabolites (Figure 2) indicates their potential at least for the upper part of the IBU biodegradation pathway.

The other IBU‐removing bacteria isolated in this work (LOIBU1.2) is a *P. aeruginosa* strain. In general, the *Pseudomonas* genus has been reported as an IBU degrader, for example, *Pseudomonas* sp. M20 degraded 20 mg L^−1^ after 12 h (Chen et al., 2023) or *P. citronellolis* CSW09 and *P. nitroreducens* CSW13 were able to remove 3.8 and 2 mg L^−1^ of IBU after 14 days, respectively (Aguilar‐Romero et al., 2024). In detail, *P. aeruginosa* is widely described in the literature for its degradative capacity of various persistent organic compounds, such, for example, as pyrene (Ma et al., 2013), carbazole, or phenols (Mahgoub et al., 2023). Moreover, its involvement in the biodegradation of some drugs is also known. For example, *P. aeruginosa* HJ1012 could completely degrade paracetamol as high as 2200 mg L^−1^ being able to mineralize this drug (Hu et al., 2013). Another study by Liu et al. (2022) demonstrated the ability of *P. aeruginosa* LY.1 to degrade 400 mg L^−1^ of flurbiprofen in 96 h. Interestingly, despite no study has been published showing the biodegradation of IBU with pure cultures of *P. aerugino*sa, it has been demonstrated that this species releases biosurfactants that can be used for the elimination of IBU (Jayalatha & Devatha, 2023). The biological treatment method that uses biosurfactant for pollutant removal generally undergoes two mechanisms: (i) adsorption of the pollutant on the micelle of the biosurfactant, and (ii) degradation of the pollutants in the biosurfactant micelle (Viramontes‐Ramos et al., 2010). The fact that no putative metabolites were detected in the aqueous samples from the bioremoval test with *P. aeruginosa* LOIBU1.2 could be due to complete degradation and mineralization, but also to biosorption (adsorption or absorption) mechanisms of IBU and/or its metabolites to the cells. Clarifying these hypotheses requires additional work; however, this does not invalidate potential applications in wastewater biotreatment.

In the case of the *Mycolicibacterium* genus, several authors have reported its ability to degrade aromatic compounds such as pyrene (Yang et al., 2021) and fuel oxygenates (Zsilinszky et al., 2022). Moreover, despite that articles describing this genus as having PC‐degrading capacity are not found, the strain tested in this work was previously selected for its ability to biodegrade paracetamol and hydroquinone (paper submitted to a scientific journal) and is now identified in this work for the first time as able remove IBU from solution, thus with potential application in wastewater biotreatment. Also in this case, the non‐detection of IBU metabolites could be due to mineralization or biosorption to the cells, and further studies are required to clarify this.

Regarding the in silico analysis, on one hand, the annotation of TIBU2.1 and HPB1.1 genomes revealed the presence of several genes involved in the metabolism of aromatic compounds, suggesting the presence of genetic potential in both strains to catabolize persistent organics pollutants, including IBU. Genetic studies on the IBU‐degradative pathway have been conducted in various microorganisms, such as *Rhizorhabdus wittichii* MPO218 and *Bacillus thuringiensis* B1, revealing the involvement of the catechol meta‐cleavage‐type pathway in IBU degradation (Aulestia et al., 2022; Marchlewicz et al., 2017). As referred to above, IBU undergoes successive reactions in the first steps of its degradation pathway resulting in the formation of isobutylcatechol. This compound is subsequently cleaved to produce 5‐formyl‐2‐hydroxy‐7‐methylocta‐2,4‐dienoic acid, suggesting the potential participation of meta‐cleavage enzymes in IBU biotransformation (Marchlewicz et al., 2017). Interestingly, as described in the results section, genes encoding proteins similar to the catechol dioxygenase referred to in the IBU degradation pathway were not found in the genomes of TIBU2.1 and HPB1.1, but the annotation in both strains of several genes in the catechol branch opens the hypothesis that both may have some enzyme that cuts isobutyl catechol. Moreover, Marchlewicz et al. (2017) observed that the IBU biotransformation process in bacteria involves the generation of intermediates such as 4‐hydroxyphenylpyruvate, which is further metabolized by enzymes like homogentisate 1,2‐dioxygenase, indicating the possible involvement of the homogentisate pathway annotated in the HPB1.1 strain's genome. Finally, it has been noted that the degradation of IBU in bacteria leads to the formation of intermediates such as 4‐hydroxyphenylpyruvate, which is subsequently metabolized by enzymes such as homogentisate 1,2‐dioxygenase, suggesting the potential involvement of the 4‐hydroxyphenylacetic acid catabolic pathway (Marchlewicz et al., 2017), which is annotated in the genome of TIBU2.1 strain.

On the other hand, the in silico search using enzymes reported by Aulestia et al. (2022) for the biodegradation of IBU as references to find putative homologues in the studied genomes revealed that both the HPB1.1 and the TIBU2.1 bacterial strains possess candidate genes encoding putative functional proteins for nearly all the studied IBU biodegradation pathway (Figure 5), which supports the hypothesis that both may be able to catabolize some steps of that pathway or even mineralize this drug. However, while in the strain HPB1.1 candidate homologue units (encoded by *ipfDE* genes) were found for the enzyme catabolizing the reaction that forms isobutyl catechol, the compound that undergoes ring cleavage, in the TIBU2.1 strain no homologues were found for both these genes. Interestingly, in the tests with the strain having more candidate genes for IBU‐catabolic enzymes (HPB1.1), no IBU transformation products were detected, while in the tests with the strain having fewer candidate genes (TIBU2.1) the HPLC and LC–MS analysis revealed an emerging peak of possible conjugates of IBU intermediate metabolites as the concentration of IBU dropped. These results corroborate the hypothesis that the TIBU2.1 strain is capable of at least transforming IBU into intermediate metabolic products while leaving open the hypothesis that the HPB1.1 strain is capable of mineralizing this drug or removing it (or its transformation products) by biosorption.

It is important to say that despite the described similarities with the used reference enzymes, to definitively prove the IBU catabolic functions of the identified candidate genes encoding putative functional proteins, additional work is necessary to study their expression in response to IBU, and/or evaluate the phenotypes of genetically engineered lines with the target gene knockdown or overexpressed. However, the objective of this work was not to study in depth each candidate gene identified with a possible function in IBU degradation, but rather to show the potential of new bacterial strains as good targets for future studies, as well as for applications in IBU biotreatment and bioremediation.

## CONCLUSION

Four bacterial strains (*K. pneumoniae* TIBU2.1, *K. variicola* LOIBU1.1, *P. aeruginosa* LOIBU1.2, and *M. aubagnense* HPB1.1) showing potential to biodegrade IBU were isolated from environmental and olive oil mill wastewater samples using enrichment liquid medium and selective solid medium with IBU as their sole energy and carbon source. Over 14 days, these strains exhibited IBU removal percentages from solution ranging from 60 to 100% of the initial IBU concentration (~5 mg L^−1^). These strains belong to species that had not been previously reported for IBU‐degrading capacity.

The genomes of *K. pneumoniae* TIBU2.1 and *M. aubagnense* HPB1.1 were studied more in detail due to their promising results in IBU removal from aqueous medium and novelty regarding PC‐degrading taxa. This enhances the genomic information available in bacteria with potential use for developing biotechnological tools.

Moreover, new putative orthologous genes possibly involved in IBU biodegradation were identified in these two strains. These genes are good candidates for further functional studies aiming at the discovery of catabolic genes and their use in the development of genetic tools for wastewater treatment.

## AUTHOR CONTRIBUTIONS

**Alba Lara‐Moreno:** Methodology; investigation; formal analysis; data curation; writing – original draft. **Maria Clara Costa:** Supervision; funding acquisition. **Ayleen Vargas‐Villagomez:** Investigation; methodology. **Jorge Dias Carlier:** Methodology; investigation; funding acquisition; writing – review and editing; project administration; supervision.

## CONFLICT OF INTEREST STATEMENT

The authors declare that there is no conflict of interest.

## Supporting information


SUPPLEMENTARY MATERIAL 1S:

SUPPLEMENTARY MATERIAL 2S:

SUPPLEMENTARY MATERIAL 3S:

SUPPLEMENTARY MATERIAL 4S:

SUPPLEMENTARY MATERIAL 5S:

SUPPLEMENTARY MATERIAL 6S:

SUPPLEMENTARY MATERIAL 7S:

SUPPLEMENTARY MATERIAL 8S:

SUPPLEMENTARY MATERIAL 9S:

## Data Availability

The data that support the findings of this study are available from the corresponding author upon reasonable request.
