# Supplementary figures and images for: New bacterial strains for ibuprofen biodegradation: Drug removal, transformation, and potential catabolic genes
Source: Environ Microbiol Rep. 2024 Aug 26;16(4):e13320. doi: 10.1111/1758-2229.13320 (PMC11347016; doi:10.1111/1758-2229.13320)

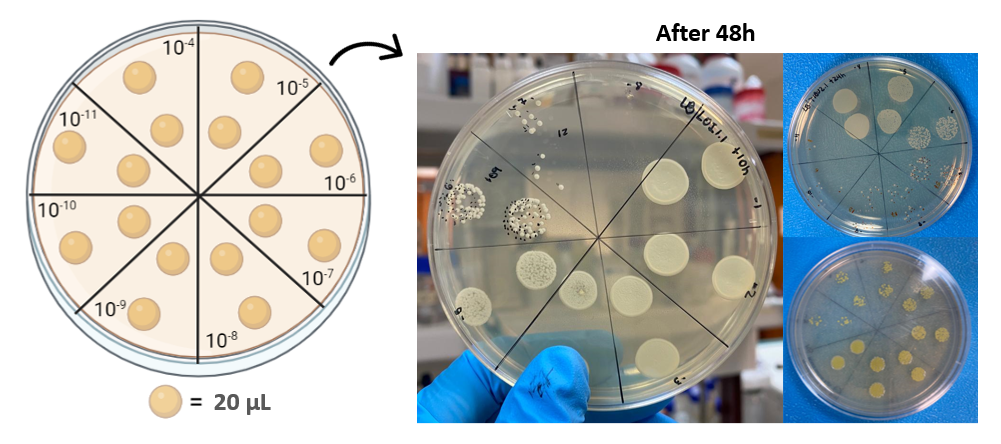


**Supplementary Material 1S.** Scheme of serial dilutions in plates.

Supplement: Supplementary file 1 — SUPPLEMENTARY MATERIAL 1S: [file EMI4-16-e13320-s004.docx]
