# Supplementary material for: New bacterial strains for ibuprofen biodegradation: Drug removal, transformation, and potential catabolic genes
Source: Environ Microbiol Rep. 2024 Aug 26;16(4):e13320. doi: 10.1111/1758-2229.13320 (PMC11347016; doi:10.1111/1758-2229.13320)
Supplement: Supplementary file 2 — SUPPLEMENTARY MATERIAL 2S: [file EMI4-16-e13320-s001.pptx]

## Slide 1
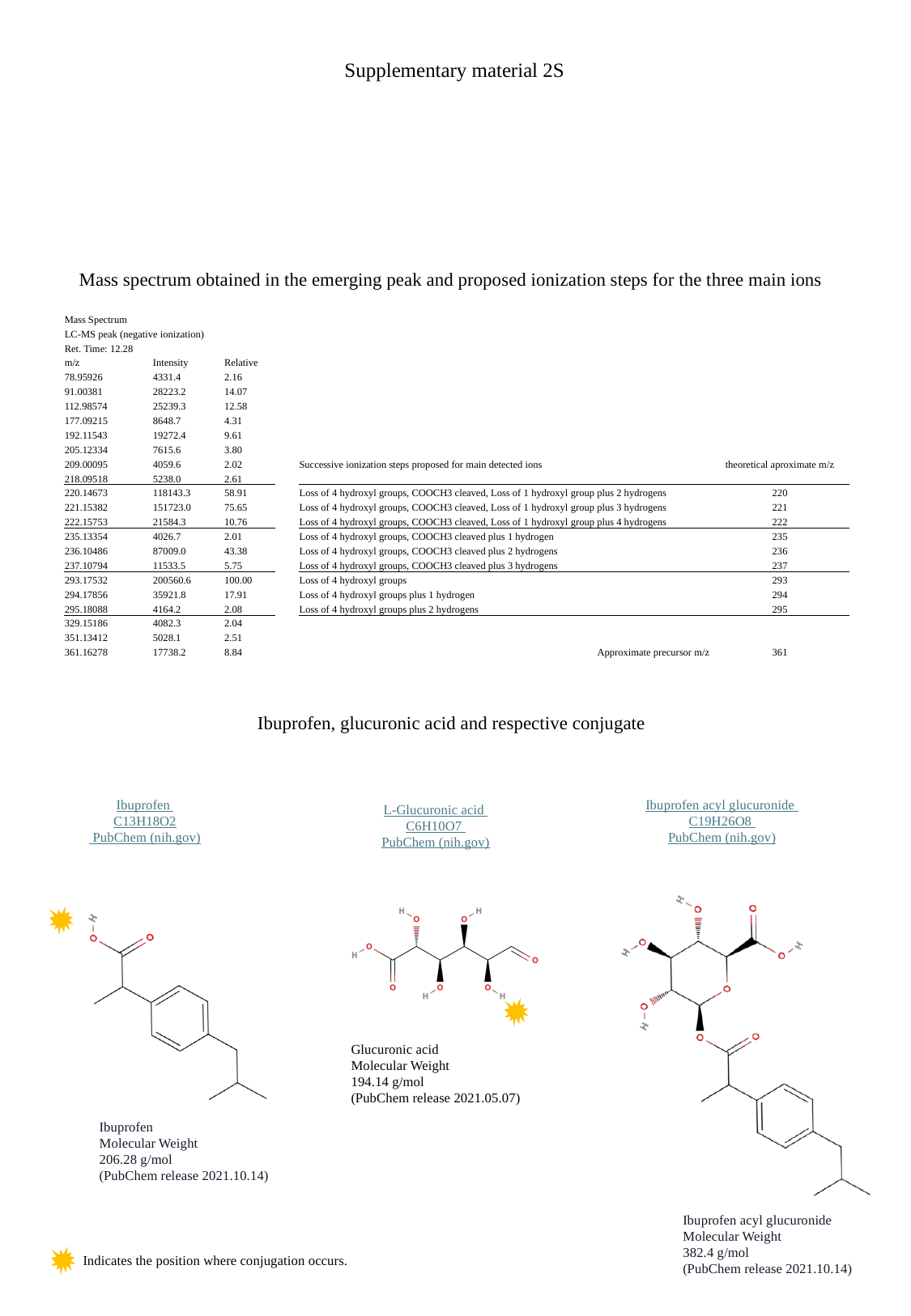

Supplementary material 2S
Mass spectrum obtained in the emerging peak and proposed ionization steps for the three main ions
| Mass Spectrum | | | | | |
| --- | --- | --- | --- | --- | --- |
| LC-MS peak (negative ionization) | | | | | |
| Ret. Time: 12.28 | | | | | |
| m/z | Intensity | Relative | | | |
| 78.95926 | 4331.4 | 2.16 | | | |
| 91.00381 | 28223.2 | 14.07 | | | |
| 112.98574 | 25239.3 | 12.58 | | | |
| 177.09215 | 8648.7 | 4.31 | | | |
| 192.11543 | 19272.4 | 9.61 | | | |
| 205.12334 | 7615.6 | 3.80 | | | |
| 209.00095 | 4059.6 | 2.02 | | Successive ionization steps proposed for main detected ions | theoretical aproximate m/z |
| 218.09518 | 5238.0 | 2.61 | | | |
| 220.14673 | 118143.3 | 58.91 | | Loss of 4 hydroxyl groups, COOCH3 cleaved, Loss of 1 hydroxyl group plus 2 hydrogens | 220 |
| 221.15382 | 151723.0 | 75.65 | | Loss of 4 hydroxyl groups, COOCH3 cleaved, Loss of 1 hydroxyl group plus 3 hydrogens | 221 |
| 222.15753 | 21584.3 | 10.76 | | Loss of 4 hydroxyl groups, COOCH3 cleaved, Loss of 1 hydroxyl group plus 4 hydrogens | 222 |
| 235.13354 | 4026.7 | 2.01 | | Loss of 4 hydroxyl groups, COOCH3 cleaved plus 1 hydrogen | 235 |
| 236.10486 | 87009.0 | 43.38 | | Loss of 4 hydroxyl groups, COOCH3 cleaved plus 2 hydrogens | 236 |
| 237.10794 | 11533.5 | 5.75 | | Loss of 4 hydroxyl groups, COOCH3 cleaved plus 3 hydrogens | 237 |
| 293.17532 | 200560.6 | 100.00 | | Loss of 4 hydroxyl groups | 293 |
| 294.17856 | 35921.8 | 17.91 | | Loss of 4 hydroxyl groups plus 1 hydrogen | 294 |
| 295.18088 | 4164.2 | 2.08 | | Loss of 4 hydroxyl groups plus 2 hydrogens | 295 |
| 329.15186 | 4082.3 | 2.04 | | | |
| 351.13412 | 5028.1 | 2.51 | | | |
| 361.16278 | 17738.2 | 8.84 | | Approximate precursor m/z | 361 |
Ibuprofen, glucuronic acid and respective conjugate
Ibuprofen acyl glucuronide
C19H26O8
PubChem (nih.gov)
Ibuprofen
C13H18O2
 PubChem (nih.gov)
L-Glucuronic acid
C6H10O7
PubChem (nih.gov)
Glucuronic acid
Molecular Weight
194.14 g/mol
(PubChem release 2021.05.07)
Ibuprofen
Molecular Weight
206.28 g/mol
(PubChem release 2021.10.14)
Ibuprofen acyl glucuronide
Molecular Weight
382.4 g/mol
(PubChem release 2021.10.14)
Indicates the position where conjugation occurs.

## Slide 2
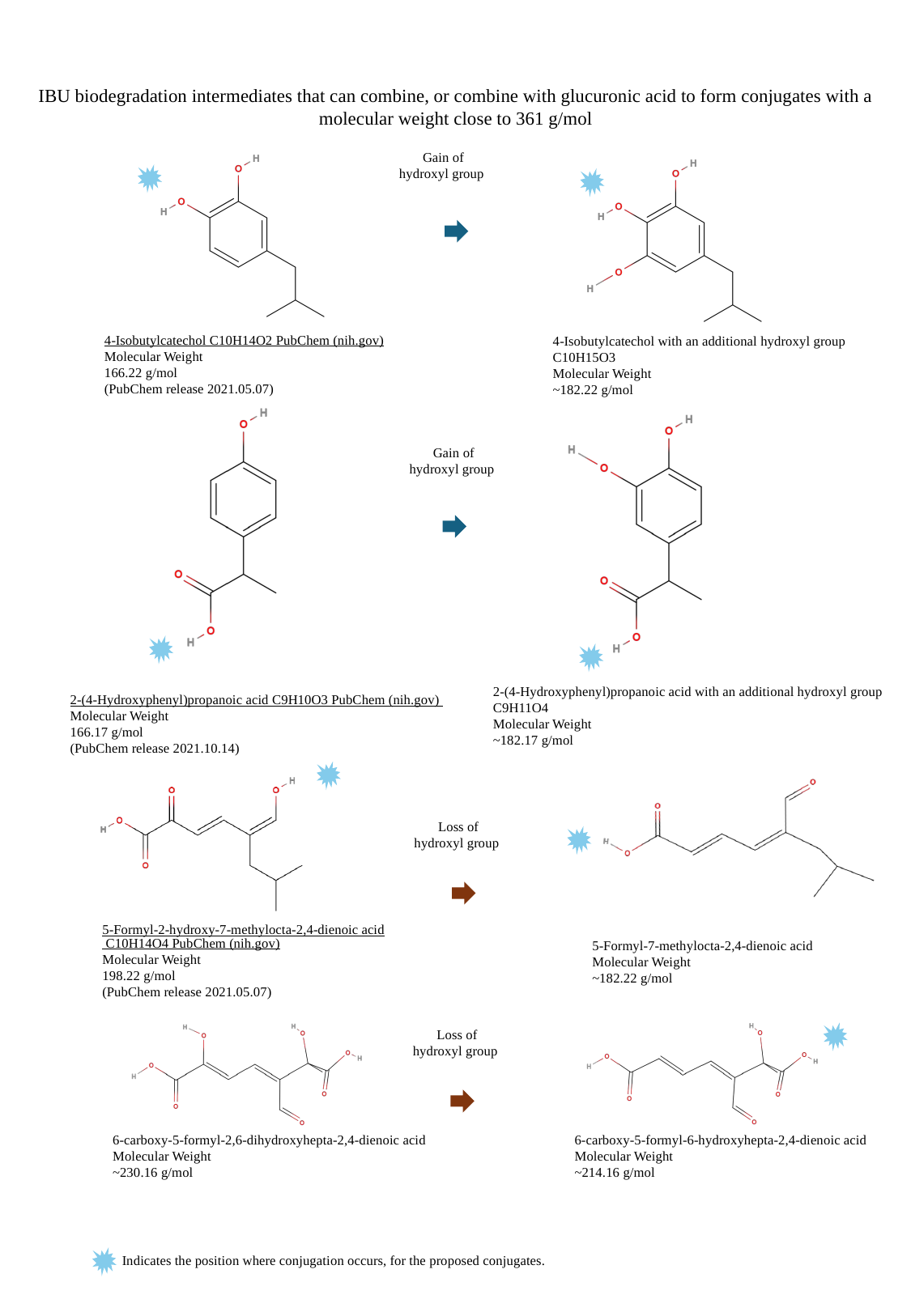

IBU biodegradation intermediates that can combine, or combine with glucuronic acid to form conjugates with a molecular weight close to 361 g/mol
Gain of
hydroxyl group
4-Isobutylcatechol C10H14O2 PubChem (nih.gov)
Molecular Weight
166.22 g/mol
(PubChem release 2021.05.07)
4-Isobutylcatechol with an additional hydroxyl group
C10H15O3
Molecular Weight
~182.22 g/mol
Gain of
hydroxyl group
2-(4-Hydroxyphenyl)propanoic acid with an additional hydroxyl group
C9H11O4
Molecular Weight
~182.17 g/mol
2-(4-Hydroxyphenyl)propanoic acid C9H10O3 PubChem (nih.gov)
Molecular Weight
166.17 g/mol
(PubChem release 2021.10.14)
Loss of
hydroxyl group
5-Formyl-2-hydroxy-7-methylocta-2,4-dienoic acid C10H14O4 PubChem (nih.gov)
Molecular Weight
198.22 g/mol
(PubChem release 2021.05.07)
5-Formyl-7-methylocta-2,4-dienoic acid
Molecular Weight
~182.22 g/mol
Loss of
hydroxyl group
6-carboxy-5-formyl-6-hydroxyhepta-2,4-dienoic acid
Molecular Weight
~214.16 g/mol
6-carboxy-5-formyl-2,6-dihydroxyhepta-2,4-dienoic acid
Molecular Weight
~230.16 g/mol
Indicates the position where conjugation occurs, for the proposed conjugates.

## Slide 3
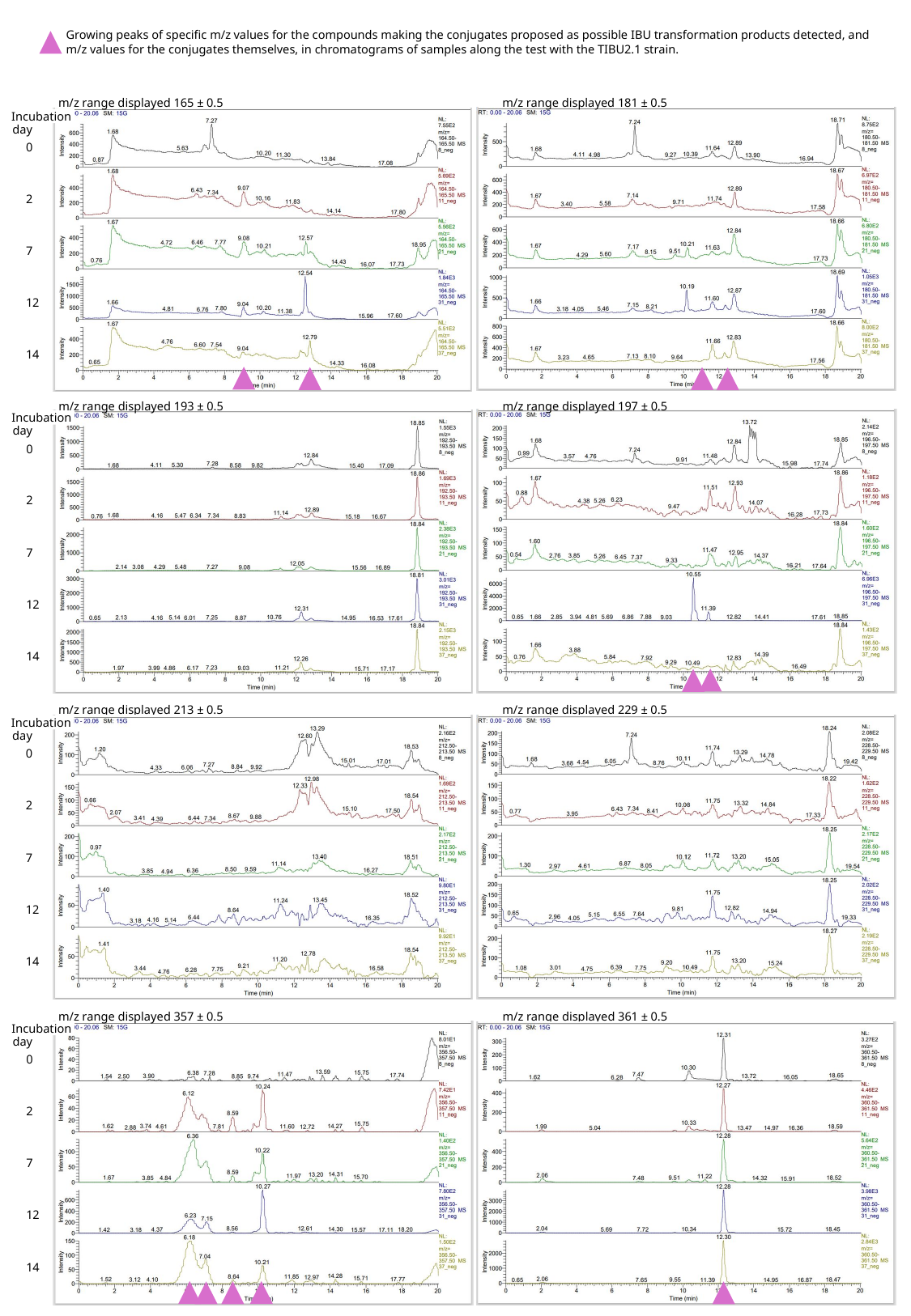

Growing peaks of specific m/z values for the compounds making the conjugates proposed as possible IBU transformation products detected, and m/z values for the conjugates themselves, in chromatograms of samples along the test with the TIBU2.1 strain.
m/z range displayed 165 ± 0.5
m/z range displayed 181 ± 0.5
Incubation
day
0
2
7
12
14
m/z range displayed 193 ± 0.5
m/z range displayed 197 ± 0.5
Incubation
day
0
2
7
12
14
m/z range displayed 213 ± 0.5
m/z range displayed 229 ± 0.5
Incubation
day
0
2
7
12
14
m/z range displayed 357 ± 0.5
m/z range displayed 361 ± 0.5
Incubation
day
0
2
7
12
14
