# Supplementary material for: New bacterial strains for ibuprofen biodegradation: Drug removal, transformation, and potential catabolic genes
Source: Environ Microbiol Rep. 2024 Aug 26;16(4):e13320. doi: 10.1111/1758-2229.13320 (PMC11347016; doi:10.1111/1758-2229.13320)
Supplement: Supplementary file 3 — SUPPLEMENTARY MATERIAL 3S: [file EMI4-16-e13320-s006.docx]

**Supplementary Material 3S.** Chromatograms and absorption spectrum detected by HPLC.

**IBU: a) Chromatogram of IBU detected at 204nm and b) absorption spectrum by HPLC.**


**TIBU2.1 strain: IBU solubilized in the aqueous Mineral Salt Medium (MSM) analyzed by HPLC in the presence of TIBU2.1. a) Chromatogram of unknown metabolite detected at 224nm, and b) absorption spectrum of 3,06 min metabolite.**

**LOIBU1.1 strain: IBU solubilized in the aqueous Mineral Salt Medium (MSM) analyzed by HPLC in the presence of LOIBU1.1. a) Chromatogram of unknown metabolite detected at 224nm, and b) absorption spectrum of 3,09 min metabolite.**
