# Supplementary material for: New bacterial strains for ibuprofen biodegradation: Drug removal, transformation, and potential catabolic genes
Source: Environ Microbiol Rep. 2024 Aug 26;16(4):e13320. doi: 10.1111/1758-2229.13320 (PMC11347016; doi:10.1111/1758-2229.13320)
Supplement: Supplementary file 4 — SUPPLEMENTARY MATERIAL 4S: [file EMI4-16-e13320-s007.docx]

**Supplementary Material 4S****.** Growth curve in LB medium of TIBU2.1, LOIBU1.1, LOIBU1.2 and *M.* *aubagnense* HPB1.1 the latter adapted from Ismail, (2022).
