# Supplementary material for: New bacterial strains for ibuprofen biodegradation: Drug removal, transformation, and potential catabolic genes
Source: Environ Microbiol Rep. 2024 Aug 26;16(4):e13320. doi: 10.1111/1758-2229.13320 (PMC11347016; doi:10.1111/1758-2229.13320)
Supplement: Supplementary file 5 — SUPPLEMENTARY MATERIAL 5S: [file EMI4-16-e13320-s002.docx]

**A1) TIBU2.1 strain**

Identities:146/544(27%), Positives:249/544(45%), Gaps:58/544(10%)

Query 5 DLVKRCARNYPTKTAYLCGERSRSWREMDQRSDRFGVALQQ-LGHRPGEAVAILTQESIE 63

+L + Y + A++ ++R++++RS F LQ+ LG + G+ VA++ ++

Sbjct 3199605 ELFEHATTRYADQPAFINMGEVMTYRKLEERSRAFAAYLQEGLGLQKGDRVALMMPNLLQ 3199426

Query 64 VYEHFFACMKIAAPRVGLNTGYVWPEMLHVLKDSEVKFLLL-------------DTRCRH 110

F ++ V +N Y E+ H L DS +++ T+ +H

Sbjct 3199425 YPVALFGILRAGMIVVNVNPLYTPRELEHQLNDSGAAAIVIVSNFAHTLEKVVAKTQVQH 3199246

Query 111 LLAERLG-ELKALGITLIGYGAGHGLER-----------DYESLLATAEGEPHW-PALAP 157

++ R+G +L TL+ + + ++R + S L + P +

Sbjct 3199245 VILTRMGDQLSTAKGTLVNFVVKY-IKRLVPKYHLPDAISFRSALQHGYRMQYVKPEIVA 3199069

Query 158 DDILFVSY**T**SGTTGVPKGVMLTQEGGVNCILHSLISFGFGPDDVWYMPAASAWVVVIL-- 215

+D+ F+ Y**T** GTTGV KG MLT + + ++ +GP + +VV L

Sbjct 3199068 EDLAFLQY**T**GGTTGVAKGAMLTHRNMLANL--EQVNATYGP----LLHRGKEFVVTALPL 3198907

Query 216 -NAFGLGNGMTTVIPDGGYQL--------QAYLRDIERFRVTVGML**V**P**TM**LQR**A**IVEIQT 266

+ F L I GG L ++++ ++ T **V** **T+** **A**++ +

Sbjct 3198906 YHIFALTMNCLLFIELGGQNLLITNPRDIPGLVKELAKYPFTAMTG**V**N**TL**FN-**A**LLNNKE 3198730

Query 267 NPVYDLSSLRMVVY**G**SSPATPKLIRDARATFKGIKLL**QAYAMT**EATGGWISYLTDADHEH 326

D SSL + **G** P + + + G LL**+ Y +T**E + D D+

Sbjct 3198729 FQQLDFSSLHLSAG**G**GMPVQ-QAVAERWVKLTGQYLL**EGYGLT**ECSPLVSVNPHDIDYHS 3198553

Query 327 ALREEIELLKSVGRIGIHYDCSIRDESGQPVPIGQSGEIWLRGNTMMKGYRNLPEATAEA 386

S+G + + D+ V GQ GE+ ++G +M GY P+AT E

Sbjct 3198552 G---------SIGLPVPSTEAKLVDDDDNEVAPGQPGELCIKGPQVMLGYWQRPDATDEI 3198400

Query 387 MPDGWLRTN**D**IGRLDERGYLY**L**LD**R**QKFLIIT**GAVN**VFPTTVEAILvehpaveevavvgv 446

+ DGWL T **D**I +DE G+L **+**+D**R**+K +I+ NV+P +E ++++H V EVA +GV

Sbjct 3198399 IKDGWLHTG**D**IAVMDEEGFLR**I**VD**R**KKDMILV**SGFN**VYPNEIEDVVMQHSGVLEVAAIGV 3198220

Query 447 phpeWGEAVVAVVVRKPSHRDVTVQALIDFCHGKLSR**P**ETPKHVVFVDELPKTSNAKLKK 506

P GEAV VV+K + +T +ALI FC L+ + PK V F DELPK++ K+ +

Sbjct 3198219 PSGSSGEAVKIFVVKKDA--ALTEEALITFCRRHLTG**Y**KVPKLVEFRDELPKSNVGKILR 3198046

Query 507 GELK 510

EL+

Sbjct 3198045 RELR 3198034

**A2) HPB1.1 strain**

Identities:177/522(34%), Positives:260/522(49%), Gaps:39/522(7%)

Query 7 VKRCARNYPTKTAYLCGERSRSWREMDQRSDRFGVALQQLGHRPGEAVAILTQESIEVYE 66

V A P A R+ +W + + R R AL G + G+ VA L + E

Sbjct 1105796 VAHWAATKPDDEAITYLNRTWTWSQWNDRVRRLAGALSAWGVKRGDVVAFLDKNHPACVE 1105975

Query 67 HFFACMKIAAPRVGLNTGYVWPEMLHVLKDSEVKFLLLDTRCR---HLLAERLGELKALG 123

A + A +N E+ VL D K L++ R +A++L ++

Sbjct 1105976 LTLAAASLGAANAIINFRLAADELDFVLNDCGAKVLVVGAELRPAVDNIADKLTHVEH-- 1106149

Query 124 ITLIGYGAGHGLERDYESLLATAEGEPHWPALAPDDILFVSY**T**SGTTGVPKGVMLTQEGG 183

I + G G E YE+LLA A P + PDD+ + Y**+**SGTTG PKGV LTQ

Sbjct 1106150 IVSVTPEGGEGDE--YEALLAGATAVDRSPDVQPDDVCIIMY**S**SGTTGRPKGVALTQ--- 1106314

Query 184 VNCILHSLISFG---FGPDD--VWYMP----A**AS**AWVVVILNAFGLGNGMTTVI---PDG 231

N I H++ +F PDD + MP **S**+++ +GL NG+ + + DG

Sbjct 1106315 ANMIAHTINAFDGWESSPDDKNLVAMPLFHVG**GS**SYM-----QYGLHNGVPSYLTRDADG 1106479

Query 232 GYQLQAYLRDIERFRVTVGML**V**P**TM**LQR**A**IVEIQTNPVYDLSSLRMVVY**GS**SPATPKLIR 291

L R L**V**P **+**L + ++E + V S+L+ Y**G+**SP L+R

Sbjct 1106480 ASLAGGILVGANRT-----FL**V**P**AV**LAK**-**VLETGPDAVKLFSALKTYAY**GA**SPMPLPLLR 1106641

Query 292 DARATFKGIKLL**QAYAMT**EATGGWISYLTDADHEHALREEIELLKSVGRIGIHYDCSIRD 351

A + + +**Q Y +T**E G +S L DH + E L S G + + + +

Sbjct 1106642 QALQAWPETEFI**QVYGLT**EVCG-VVSRLMPEDHRA---DNEERLVSAGVLIPEAEVRVVN 1106809

Query 352 -ESGQPVPIGQSGEIWLRGNTMMKGYRNLPEATAEAM-PDGWLRTN**D**IGRLDERGYLY**L**L 409

E+ P G+ GE+W R T+MK Y N P+ATAEA+ PDGW RT **D**IGR+D GY++**+**

Sbjct 1106810 PETLDDAPAGEQGELWFRTPTLMKEYLNRPDATAEAITPDGWFRTG**D**IGRVDADGYIF**V**E 1106989

Query 410 **D**RQKFLIIT**GAVN**VFPTTVEAILvehpaveevavvgvphpeWGEAVVAVVVRKPSHRDVT 469

**D**R K +II+**G N**++ VE +L EHPAV EVA++GVP +WGE+V AVV + S V+

Sbjct 1106990 **D**RLKDMIIS**GGEN**IYSIEVERVLAEHPAVSEVAIIGVPDEKWGESVKAVVTLEGSGDGVS 1107169

Query 470 VQALIDFCHGKLSR**P**ETPKHVVFVDELPKTSNAKLKKGELKK 511

LI F +L+ + PK + FVD++P+ K+ K EL+K

Sbjct 1107170 DADLIAFARERLAA**Y**KCPKTIDFVDDMPRNPTGKILKKELRK 1107295

**B)**


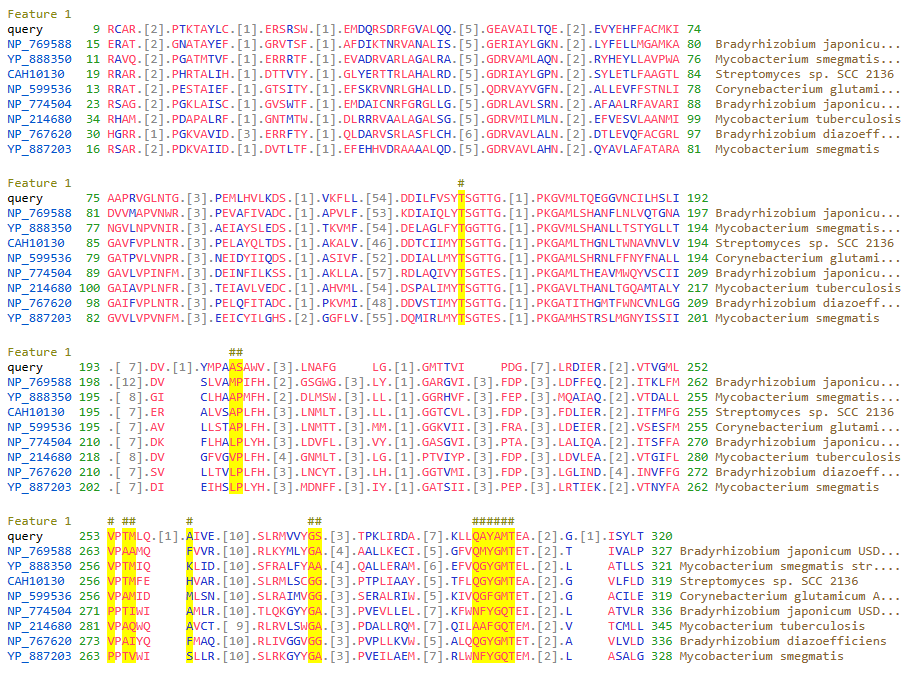


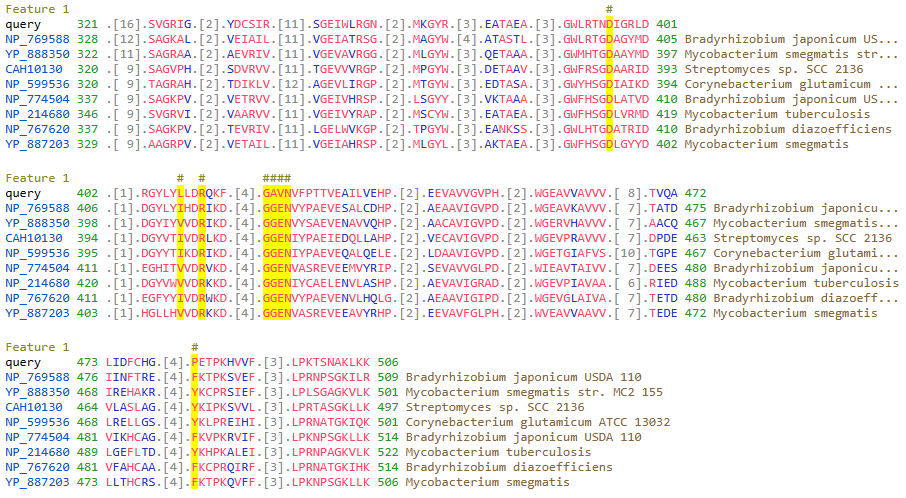


**Supplementary Material 5S.** A) Sequence alignment between AMP-binding protein (IpfF, Accession number: WP_208634566.1) from *Sphingomonadaceae* (Query) and studied protein from *K.pneumoniae* TIBU2.1 (Sbjct) (A1) or *M. aubagnense* HPB1.1 (A2). Amino acids in red correspond to active sites involved in the feature, highlighted in yellow correspond to the active sites common to both species, and highlighted in green correspond to active sites with possible amino acids for other species, B) Sequence alignment between studied candidate AMP-binding protein from TIBU2.1 (query) and AMP-binding protein from different strains using the Conserved Domain Database (CDD). Red indicates highly conserved, and blue indicates less conserved. Unaligned residues are shown in grey. Amino acids highlighted in yellow and hash marks (#) correspond with active sites involved in the feature.
