# Supplementary material for: New bacterial strains for ibuprofen biodegradation: Drug removal, transformation, and potential catabolic genes
Source: Environ Microbiol Rep. 2024 Aug 26;16(4):e13320. doi: 10.1111/1758-2229.13320 (PMC11347016; doi:10.1111/1758-2229.13320)
Supplement: Supplementary file 6 — SUPPLEMENTARY MATERIAL 6S: [file EMI4-16-e13320-s008.docx]

**A1) TIBU2.1 strain**

Identities:121/410(30%), Positives:197/410(48%), Gaps:40/410(9%)

Query 8 TITRKAPDVDVGALIERD----RIHGSLYANESIFELEMKKIFYDGWVFVGHDSEVPTAG 63

T++ ++ +++R+ R H S++ ++ +F+ EMK IF WVF+ H+S++ G

Sbjct 2726655 TLSTLKDKINNALVVDRENHIYRCHRSIFTDQQLFDFEMKHIFEGNWVFLAHESQIAEPG 2726476

Query 64 EYVRRTLGREEVLMVRQRDSSIAVIANRCAHRGNMMCIANHGKEKYFTCTYHGWVYDLAG 123

+Y TLGR+ V++ R + + + + N CAHRG M+C G + FTC +HGW + G

Sbjct 2726475 DYYTLTLGRQPVIITRDKKNELHALINSCAHRGAMLCRRKTGNKNSFTCPFHGWTFSNNG 2726296

Query 124 NL---KDVP---YPGGFDKDKSELKLQPL-RTEVYRGFVFATFNASAPPLMEQLGRGKIL 176

L KD YP F +D S LQ L R + YRGF+F + A PL LG +

Sbjct 2726295 KLLKAKDESTGGYPPSFKQDGSH-DLQKLPRFQSYRGFLFGSLKADVQPLEAYLGETCKI 2726119

Query 177 IDRACDMSPTGRLQLTAGWTKQRFGANWKMLPE**ND**T**DG**Y**HV**NDV**H**ASFAQVID-SHYDSA 235

ID D +P G L++ G + + NWK+ E**N** **DG**Y**HV**+ V**H** ++A + +Y++

Sbjct 2726118 IDLIVDQAPEG-LEVLKGSSSYVYEGNWKLGAE**NG**A**DG**Y**HV**SVV**H**WNYASTMSRRNYEAE 2725942

Query 236 AIAAEDS---LRSQAKDWG--NGHTELYLSPTYTEYLKWFNTTPNRFPEYI--AQMKAAY 288

A D+ +S +G NGH L+ P P Y +++A +

Sbjct 2725941 GTHAVDANGWSKSLGGGYGFDNGHMLLWTRA----------LNPEVRPVYAHRERLQAEF 2725792

Query 289 GEEKGDNILRD**GP**P**H**ATIFPNL**F**L**-**--**-**-GEMNI**I**IFLPINAHECVQWHTPMLLEGAPDE 343

GE + D ++ + **+** ++PN+**+**L ++ +**I** + ++ E W +G D+

Sbjct 2725791 GERRADQMVNE**-T**R**N**LCLYPNV**Y**L**M**DQ**F**STQIRV**I**RPIAVDKTEVTIW--CFAPKGESDQ 2725621

Query 344 VNQRIIRN**S**EA**AM**GPSAF**LL**AD**D**-SVISERQQIALRDRADWLDVSRGLNR 392

IR E S D**D** S Q+ L + W D+SRG R

Sbjct 2725620 ARALRIRQ**Y**ED**FF**NVSGM**GT**PD**D**LEEFSACQRGYLGENLPWSDLSRGALR 2725471

**A2) HPB1.1 strain**

Identities:185/412(45%), Positives:251/412(60%), Gaps:10/412(2%)

Query 21 LIERDRIHGSLYANESIFELEMKKIFYDGWVFVGHDSEVPTAGEYVRRTLGREEVLMVRQ 80

LI RDR+HGSLY + SIF E+ KI+Y WVFVGH+SEV +YVR+ LG ++V+M R

Sbjct 742648 LIHRDRVHGSLYTDPSIFAEELAKIWYRTWVFVGHESEVAQPNDYVRKKLGPQDVIMTRD 742827

Query 81 RDSSIAVIANRCAHRGNMMCIANHGKEKYFTCTYHGWVYDLAGNLKDVPYPGGFDKDKSE 140

R+ I ++ NRCAHRGN +C G F C YHGW + G L P+ G+ + K +

Sbjct 742828 REGQIHLLLNRCAHRGNQVCDDAKGNSGTFRCPYHGWTFRNTGELIGFPFFKGYGERKLD 743007

Query 141 LKLQPL-RTEVYRGFVFATFNASAPPLMEQLGRGKILIDRACDMSPTGRLQLTAGWTKQR 199

L + + R + Y GFVF +F A P L+E LG IDR +SP GR++LTAGW +

Sbjct 743008 LAMGRVPRVDSYGGFVFGSFAADGPSLVEHLGDATGEIDRLTRLSPEGRVELTAGWLQHT 743187

Query 200 FGANWKMLPE**ND**T**DG**Y**HV**NDV**H**ASFAQVIDSHYDSAAIAAEDSLRSQAKDWGNGHTELYL 259

ANWK+L E**N+**T**DG**Y**H** V**H** S V S + DS + +D GNGH+E L

Sbjct 743188 TRANWKLLAE**NE**T**DG**Y**HP**QFV**H**GSIFGVTGS---TIGPLYSDSSTAVTRDLGNGHSENDL 743358

Query 260 SPTYTEY---LKWFNTTPNRFPEYIAQMKAAYGEEKGDNILRD**G**P**PH**ATIFPNL**F**L**G**EM**N** 316

P + ++ ++WF TT +R P+Y+A ++AA G + + IL +**G** **PH** IFPNL**F**+ E+

Sbjct 743359 RPEFRKFAEPMRWFGTTESRVPDYVAAIRAARGAD-AERILIE**G**A**PH**VMIFPNL**F**I**A**EI**Q** 743535

Query 317 I**I**IFLPINAHECVQWHTPMLLEGAPDEVNQRIIRN**S**EA**AM**GPSAF**LL**AD**D**SVISERQQIA 376

+ P++ ECVQ+ T + L GAP E+N+R++ **++**GP+ **LL**AD**D**+ + ER Q

Sbjct 743536 V**F**NIQPVSVDECVQYATAVQLAGAP-ELNRRMVSQ**C**VG**SV**GPAGM**LL**AD**D**TEMYERNQHG 743712

Query 377 LRD-RADWLDVSRGLNREHVDEMGVVVGHVTDECTNRGFWQHYKKVMTAPSP 427

L +WLDV RG+NRE VDE G VG TDE RGFW HYK +M P P

Sbjct 743713 LAALNPEWLDVRRGINRETVDERGHPVGSATDETGMRGFWSHYKNLMETP*P 743868

**B)**


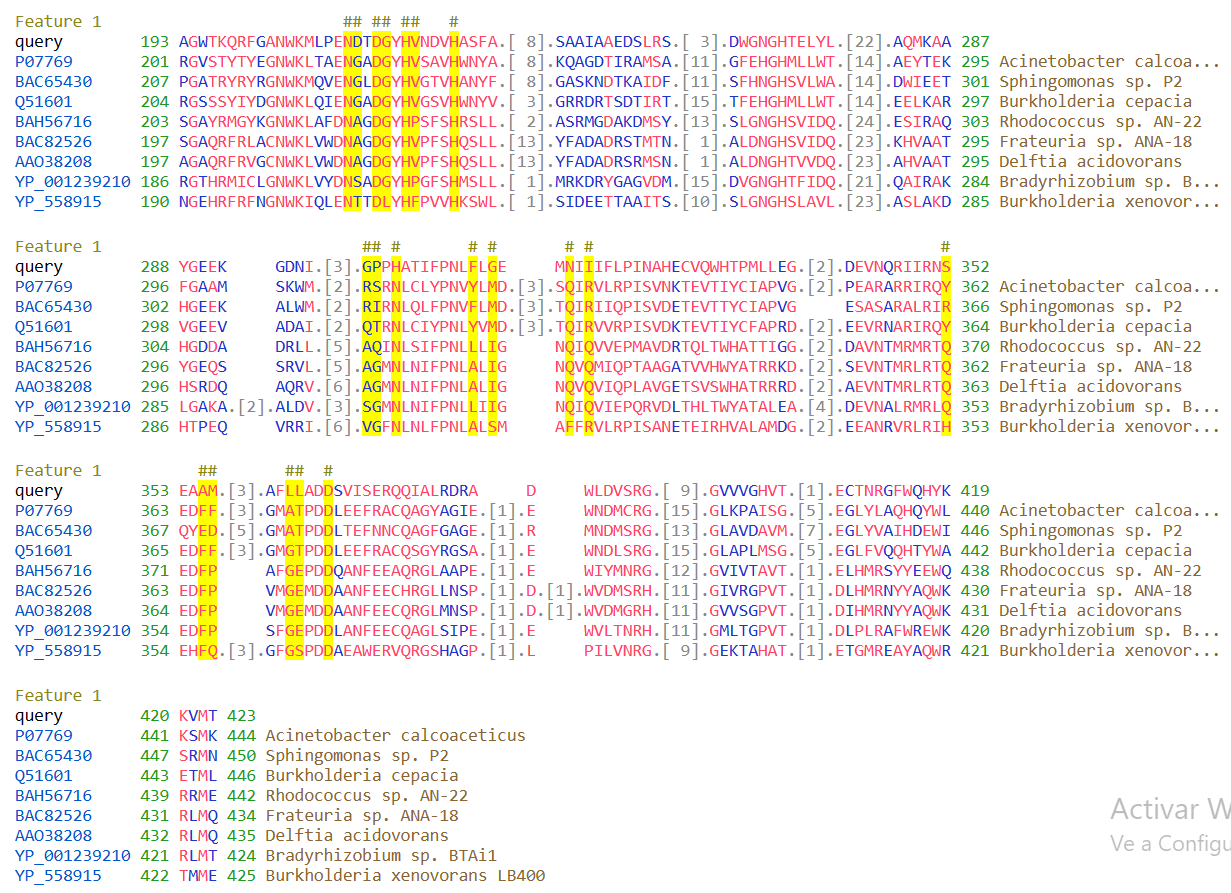


**Supplementary Material 6S.** A) Sequence alignment between aromatic ring-hydroxylating dioxygenase α subunit protein (IpfA, Accession number: WP_208634570.1) from *Rhizorhabdus wittichii* (Query) and studied protein from *K.pneumoniae* TIBU2.1 (Sbjct) (A1) or *M. aubagnense* HPB1.1 (A2). Amino acids in red correspond to active sites involved in the feature, highlighted in yellow correspond to the active sites common to both species, and highlighted in green correspond to active sites with possible amino acids for other species, B) Sequence alignment between studied candidate aromatic ring-hydroxylating dioxygenase α subunit from TIBU2.1 (query) and aromatic ring-hydroxylating dioxygenase subunit alpha protein from different strains using the Conserved Domain Database (CDD). Red indicates highly conserved, and blue indicates less conserved. Unaligned residues are shown in grey. Amino acids highlighted in yellow and hash marks (#) correspond with active sites involved in the feature.
