# Supplementary material for: New bacterial strains for ibuprofen biodegradation: Drug removal, transformation, and potential catabolic genes
Source: Environ Microbiol Rep. 2024 Aug 26;16(4):e13320. doi: 10.1111/1758-2229.13320 (PMC11347016; doi:10.1111/1758-2229.13320)
Supplement: Supplementary file 7 — SUPPLEMENTARY MATERIAL 7S: [file EMI4-16-e13320-s003.docx]

**A1) TIBU2.1 strain**

Identities:24/83(29%), Positives:36/83(43%), Gaps:7/83(8%)

Query 29 FAVYDVGGTYYVTDNI**C**T**H**GNAMLTDGYQDGGTIE**C**PF**H**GGAF-------DIASGAATVF 81

+Y + G V ++**C** **H** LT G+ + I **C**P+**H**G F I S

Sbjct 2494748 LVIYRIKGQVVVARDV**C**P**H**RGVPLTLGFHEEEGIV**C**PY**H**GLRFGEDGRCNRIPSSPGQPI 2494569

Query 82 PCQIPLKTYSVEVDDGWIAIRLA 104

P ++ L +++VE G I LA

Sbjct 2494568 PAKLHLTSFAVEERYGLIWTCLA 2494500

**A2) HPB1.1 strain**

Identities:34/99(34%), Positives:46/99(46%), Gaps:2/99(2%)

Query 10 VDEVKEGEPVAAHVAGLPPFAVYDVGGTYYVTDNI**C**T**H**GNAMLTDGYQDGGTIE**C**PF**H**GG 69

+D + EGE VA GL A+ + G Y DN **C** **H** L G + I **C**P+**H**G

Sbjct 2385186 IDTLDEGE-VAMCPVGLKTVALTKLEGRYGAIDNR**C**P**H**QGGPLGQGTLENDKIR**C**PW**H**GF 2385010

Query 70 AFDIASGAATVFPCQIPLKTYSVEVDDGWIAIRLASPEA 108

FD +G A P ++TY VEV + + + P A

Sbjct 2385009 DFDPFTGEAAGGP-DFNVRTYPVEVREDGVYVGTPPPAA 2384896

**B)**


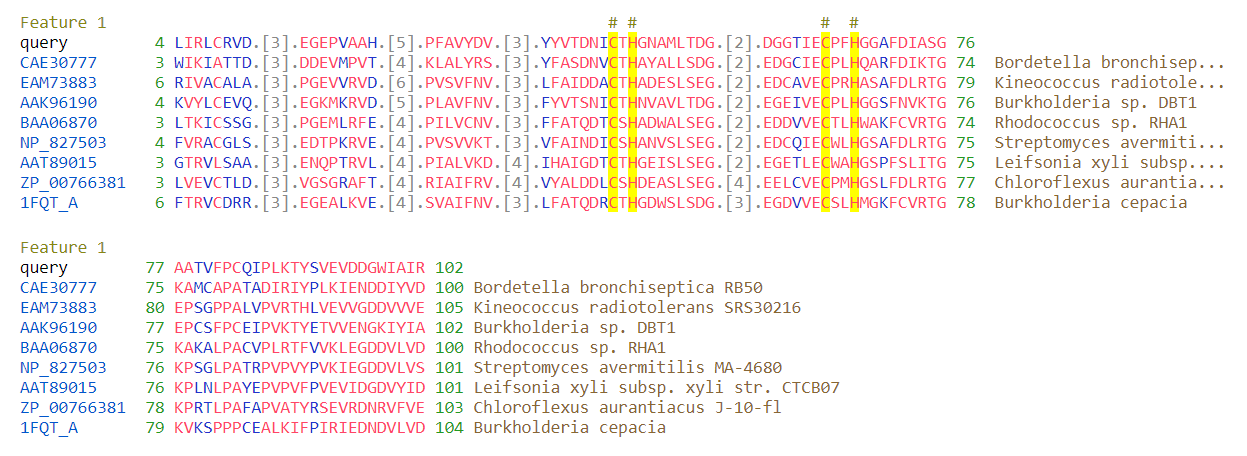


**Supplementary Material 7S.** A) Sequence alignment between non-heme iron oxygenase ferredoxin protein (IpfI, Accession number: WP_185210326.1) from *Sphingomonadaceae* (Query) and studied protein from *K. pneumoniae* TIBU2.1 (Sbjct) (A1) or *M. aubagnense* HPB1.1 (A2). Amino acids in red correspond to active sites involved in the feature, highlighted in yellow correspond to the active sites common to both species, B) Sequence alignment between studied candidate iron oxygenase ferredoxin protein from TIBU2.1 (query) and iron oxygenase ferredoxin protein from different strains using the Conserved Domain Database (CDD). Red indicates highly conserved, and blue indicates less conserved. Unaligned residues are shown in grey. Amino acids highlighted in yellow and hash marks (#) correspond with active sites involved in the feature.
