# Supplementary material for: New bacterial strains for ibuprofen biodegradation: Drug removal, transformation, and potential catabolic genes
Source: Environ Microbiol Rep. 2024 Aug 26;16(4):e13320. doi: 10.1111/1758-2229.13320 (PMC11347016; doi:10.1111/1758-2229.13320)
Supplement: Supplementary file 8 — SUPPLEMENTARY MATERIAL 8S: [file EMI4-16-e13320-s005.docx]

**A1) TIBU2.1 strain**

Identities:197/488(40%), Positives:288/488(59%), Gaps:12/488(2%)

Query 1 MPEIRHFIDGDYRASISGKTFPKHRPYDGRHILDVNEGGREEVDAAVSAARTALQGPWAT 60

M +I H+I+G A F P G + +V GG E++ AV+AA+ A WA

Sbjct 701653 MKKINHWINGKNVAG--ADYFHTTNPATGEVLAEVASGGEAEINQAVAAAKEAFP-KWAN 701483

Query 61 ISVDDRADLMRAIADGITKRFDDFVSAEMNDTGQPISAMRHAFVPRGAANFNAFADLVKS 120

+ + +RA LMR + D I + + S E DTG PI ++ +PR + NF FA++ +

Sbjct 701482 LPMKERARLMRRLGDLIDQNVPEIASMETADTGLPIHQTKNVLIPRASHNFEFFAEVCQQ 701303

Query 121 VSTECFHTETPDGRGALNYAIRKPKGVIGVISPW**N**APFLLMTWKVAPALACGNTVVVKPS 180

++ + + P LNY + +P GV ++SPW**N** PF+ TWKVAP LA GNT V+K S

Sbjct 701302 MNGKTY----PVDDKMLNYTLVQPVGVCALVSPW**N**VPFMTATWKVAPCLALGNTAVLKMS 701135

Query 181 EETPSTATLLGEVMNEVGVPKGVYNVVHGFGPDSAGAYLTEHQGVDAITFTGETRTGTAI 240

E +P TA LGE+ E G+P GV NVV G+G +AG L H V A++FTG T TG I

Sbjct 701134 ELSPLTADRLGELALEAGIPAGVLNVVQGYGA-TAGDALVRHHDVRAVSFTGGTATGRNI 700958

Query 241 MQAAARGVRDVSF**E**LGGKNSAIIFadadldaalEGLMRSVFLNT**G**QV**C**LGTERVFVERPL 300

M+ A G++ S **E**LGGK+ +IF DAD++ AL+ + ++F **G**+ **C** R+F+++ +

Sbjct 700957 MKNA--GLKKYSM**E**LGGKSPVLIFEDADIERALDAALFTIFSIN**G**ER**C**TAGSRIFIQQSI 700784

Query 301 FERFVGALGERVKSLKPGHPEGAGTSFGPLISSEHREKVLGYYrraradgaviva--ggg 358

+ FV ER L+ G P T G LIS +H EKV GY R +GA ++A

Sbjct 700783 YPEFVKRFAERANRLRVGDPTDPNTQVGALISQQHWEKVSGYIRLGIEEGATLLAGGADK 700604

Query 359 vPELPAELSGGSWVEPTIWTGLPDDHPVIEEEIFGPCCHVRPFDSEEEVIGLANNTSYGL 418

+LPA L G+++ PT+ + + V +EEIFGP + PF E E + LAN+ YGL

Sbjct 700603 PSDLPAHLKAGNFLRPTVLADVDNRMRVAQEEIFGPVACLLPFKDEAEGLRLANDVEYGL 700424

Query 419 SSAIWTQNLSRAHRMAAAIDVGITWVNSWFLRDLRTPFGGTRQSGIGREGGVHSLEFYSE 478

+S IWTQ++S+ R+A I+ G+ +VN+ +RDLR PFGG + SG GREGG +S E ++E

Sbjct 700423 ASYIWTQDVSKVLRLARGIEAGMVFVNTQNVRDLRQPFGGVKASGTGREGGEYSFEVFAE 700244

Query 479 LRNVCVKL 486

++NVC+ +

Sbjct 700243 MKNVCISM 700220

**A2) HPB1.1 strain**

Identities:186/492(38%), Positives:265/492(53%), Gaps:24/492(4%)

Query 5 RHFIDGDYRASISGKTFPKHRPYDGRHILDVNEGGREEVDAAVSAARTALQ-GPWATISV 63

+ ++DG + S+SG+ F P + I V E+V+ AV AAR A + GPW T++

Sbjct 5032267 QMYVDGQWVESVSGRRFDTVDPATEQVITTVPHSDAEDVERAVRAARRAFEHGPWPTMTP 5032088

Query 64 DDRADLMRAIADGITKRFDDFVSAEMNDTGQPISAMRHAFVPRGAANFNAFADLVKSVST 123

+R ++ IA+GIT R D F E D G+ ++ + V A F +A +

Sbjct 5032087 AERQRMIWRIAEGITARADQFAELESIDNGKSVAVAKAVDVTWAAEIFYYYAGWATKIEG 5031908

Query 124 ECFHTETPDGRGALNYA--IRKPKGVIGVISPW**N**APFLLMTWKVAPALACGNTVVVKPSE 181

P G +A +R+P GV I+PW**N** P ++ +KV PALACGNT+++KP+E

Sbjct 5031907 RTVPVSVPWAPGGKFHAFTLREPVGVCAQITPW**N**FPLVMAAFKVGPALACGNTIILKPAE 5031728

Query 182 ETPSTATLLGEVMNEVGVPKGVYNVVHGFGPDSAGAYLTEHQGVDAITFTGETRTGTAIM 241

+TP TA LL EV+ E GVP GV+N++ GFG GA L+ H GVD + FTG T G I+

Sbjct 5031727 QTPLTAVLLAEVIAEAGVPAGVFNLLTGFG--DVGAALSAHDGVDKVAFTGSTEVGKKIV 5031554

Query 242 QAAARGVRDVSF**E**LGGKNSAIIFadadldaalEGLMRSVFLNT**G**QV**C**LGTERVFVERPLF 301

AA+ ++ VS **E**LGGK+ ++FADADL+AA+ G+ N **G**Q **C** R+ VE +F

Sbjct 5031553 NAASGNLKKVSL**E**LGGKSPQVVFADADLEAAIPGVAGGFLFNH**G**QT**C**TAGTRLLVEDTIF 5031374

Query 302 ERFVGALGERVKSLKPGHPEGAGTSFGPLISSEHREKVLGYYrraradgavivagggvPE 361

+ F + E L+ G GPL+S E KV GY A GA

Sbjct 5031373 DEFTQGVAEHAAGLRIGPGLDPTNDIGPLVSREQLTKVTGYLDDGIAQGARA-------- 5031218

Query 362 LPAELSGGS-------WVEPTIWTGLPDDHPVIEEEIFGPCCHVRPFDSEEEVIGLANNT 414

LSGG +V+PT+ + D V +EEIFGP PF+ + V AN+T

Sbjct 5031217 ----LSGGGRHGDTGFYVQPTLLVDVHRDFNVYQEEIFGPVAVAVPFNRDRGVREAANDT 5031050

Query 415 SYGLSSAIWTQNLSRAHRMAAAIDVGITWVNSWFLRDLRTPFGGTRQSGIGREGGVHSLE 474

YGL+++IWT+++S AHR+A I G WVN D PFGG +QSG GRE G ++

Sbjct 5031049 PYGLAASIWTRDVSTAHRVAQQIKAGTVWVNCHNAFDTALPFGGYKQSGWGRELGEGAIA 5030870

Query 475 FYSELRNVCVKL 486

Y++ + + + L

Sbjct 5030869 EYTQTKAINIAL 5030834

**B)**


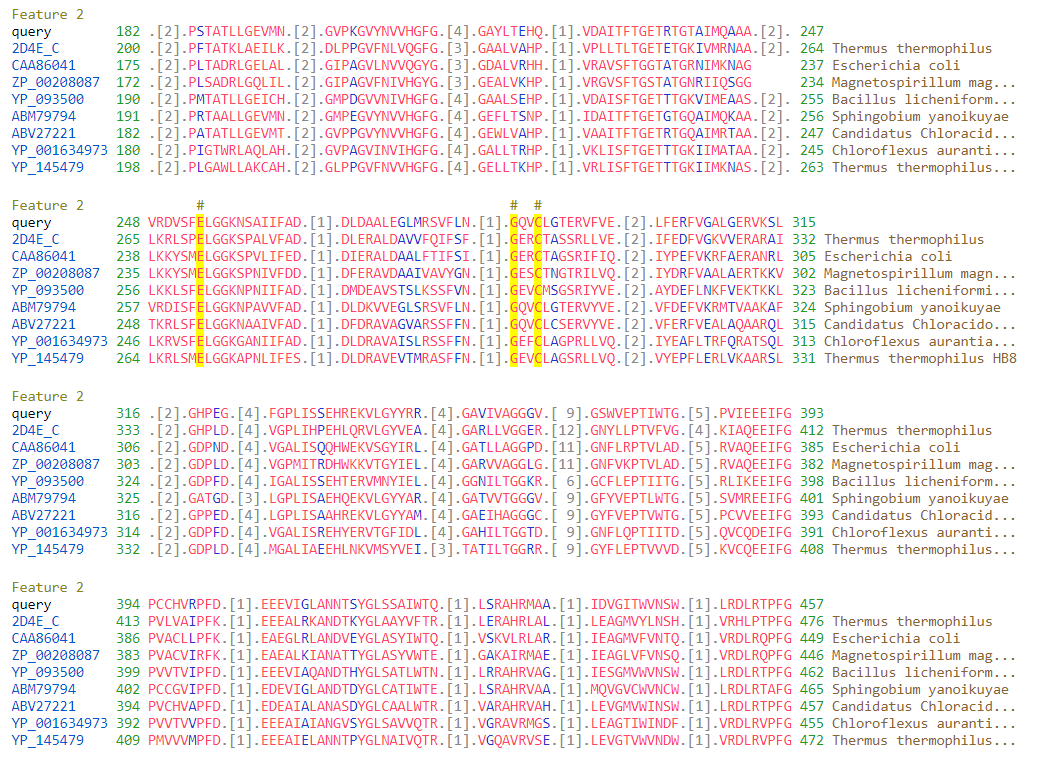


**Supplementary Material 8S.** A) Sequence alignment between 2-hydroxymuconic semialdehyde dehydrogenase protein (IpfM, Accession number: WP_208634595.1) from *Sphingomonadaceae* (Query) and studied protein from *K. pneumoniae* TIBU2.1 (Sbjct) (A1) or *M. aubagnense* HPB1.1 (A2). Amino acids in red correspond to active sites involved in the feature, highlighted in yellow correspond to the active sites common to both species, B) Sequence alignment between studied candidate 2-hydroxymuconic semialdehyde dehydrogenase protein from TIBU2.1 (query) and 2-hydroxymuconic semialdehyde dehydrogenase protein from different strains using the Conserved Domain Database (CDD). Red indicates highly conserved, and blue indicates less conserved. Unaligned residues are shown in grey. Amino acids highlighted in yellow and hash marks (#) correspond with active sites involved in the feature.
