# Supplementary material for: New bacterial strains for ibuprofen biodegradation: Drug removal, transformation, and potential catabolic genes
Source: Environ Microbiol Rep. 2024 Aug 26;16(4):e13320. doi: 10.1111/1758-2229.13320 (PMC11347016; doi:10.1111/1758-2229.13320)
Supplement: Supplementary file 9 — SUPPLEMENTARY MATERIAL 9S: [file EMI4-16-e13320-s009.docx]

1. **HPB1.1 strain**

Identities:171/453(38%), Positives:232/453(51%), Gaps:21/453(4%)

Query 12 FALEHAAGLARIAEHADFAEATRETVEAVLQGIGVLAAGEYAPTNRVGDTAGPRWID--G 69

F L + + +H FAE +R+T +AVL +A +AP N+ D P ++D G

Sbjct 2179308 FLLYEWLDVVELTKHEHFAEHSRDTFDAVLDLSADIATKRFAPHNKKAD-HNPPFVDETG 2179484

Query 70 RVEMPAAFHTAYRAFVEGGWGGIAVPTEHGGMGLPFSLAVAAMESLGTANMGFGLIHLLS 129

+V + A F G + GGM LP +A ++ AN L+

Sbjct 2179485 KVVLIDEIKQALDDFNAAGLMASSFDERVGGMQLPNVVAQSSAAFFRAANPATFSYAFLT 2179664

Query 130 FGAIHAIEVYGSDHQKAVWLPHLVNGRWNGT**M**N**LT**EPLA**GS**DVGALRTRAEQAADEGLWR 189

G + + YG+D Q W+ ++ GR+ GT**M** **L+**EP A**GS** + + T+A +AAD G +R

Sbjct 2179665 VGNANLLTEYGTDEQIDTWVRPMLEGRYFGT**M**C**LS**EPDA**GS**SLADISTKATKAAD-GTYR 2179841

Query 190 IRGQKI**F**I**T**FGECDLVENVVHLVLARTNDAPKGTKGISLFLVPKLRLDDQGRPAIPNGVH 249

+ G K+**+**I**T** G+ +L EN+VHLVLA+ G KGISLF+VPK D N V

Sbjct 2179842 VTGTKM**W**I**T**GGDHELTENIVHLVLAKVPGGGPGVKGISLFIVPKFLTD-----GTRNDVA 2180006

Query 250 CVSIEHKLGIHGSPTCVMAYGE------DGEECLGELVGEIGGGMRAMFVMMNRARLLVG 303

VS+ HK+G HG+ ++ +G+ D +G LVGE G+ MF MMN AR+ VG

Sbjct 2180007 LVSLNHKMGNHGTTNALLNFGDGSHRPTDEPGAVGYLVGEEHRGLAYMFHMMNEARVAVG 2180186

Query 304 NQGVQIAERATQQALAYAHQRIQSARA--SDPVSGAVAIIEHPDVRRMLLRMKSLTQAAR 361

+ I +L YA R Q DP V IIEH DV+RMLL KS + A

Sbjct 2180187 SMATAIGYAGYLTSLEYAKVRTQGRPVGDKDPTRSQVPIIEHADVKRMLLAQKSYVEGAL 2180366

Query 362 AL----TYacagaidmgaagddkaaaraEVLTPLAKSWASDIGCEVASLGIQVHGGMGYI 417

AL A D+A EVLTP+AKSW S E +LGIQ+HGG GY

Sbjct 2180367 ALGLYCARLVDEMRIFTGAELDEANLMLEVLTPIAKSWPSQWCLEANNLGIQIHGGYGYT 2180546

Query 418 EETGAAQHYRDAR**I**APIYEG**T**N**G**IQAADLVGRK 450

E Q YRD R**+** I+EG**T**+**G**I DL+GRK

Sbjct 2180547 TEYDVEQQYRDNR**L**NAIHEG**T**H**G**IHGIDLLGRK 2180645

**B)**


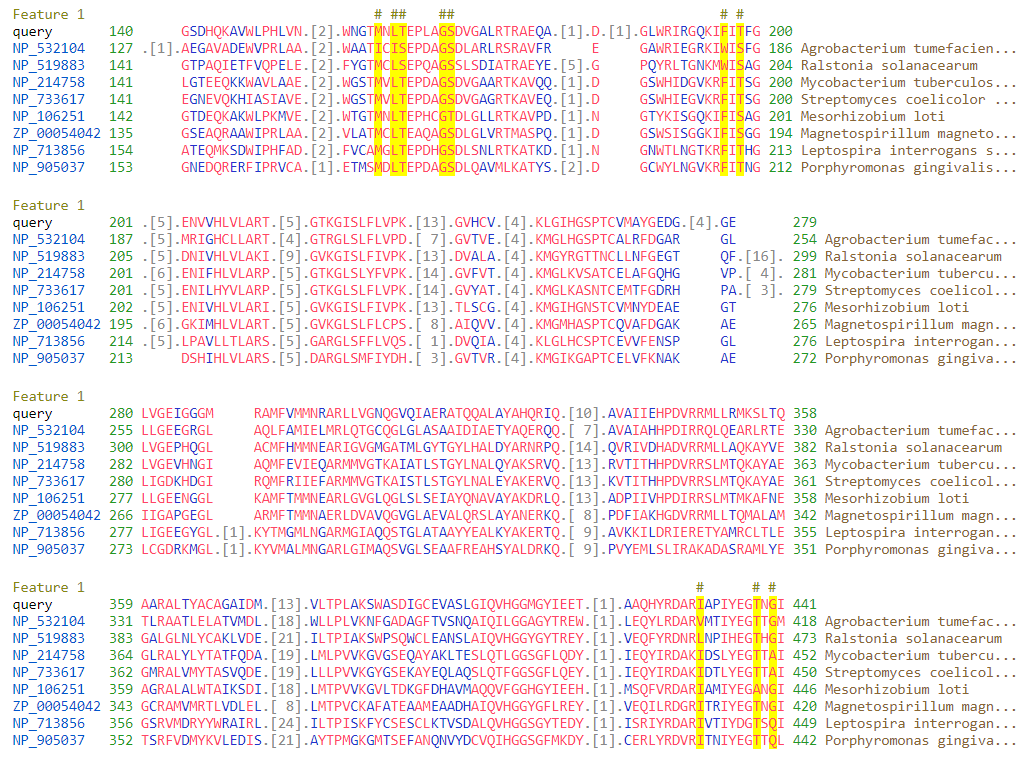


**Supplementary Material 9S.** A) Sequence alignment between acyl-CoA dehydrogenase protein (IpfT, Accession number: WP_208634517.1) from *Sphingomonadaceae* (Query) and studied protein from *M. aubagnense* HPB1.1 (Sbjct) (A1). Amino acids in red correspond to active sites involved in the feature, highlighted in yellow correspond to the active sites common to both species, B) Sequence alignment between studied candidate acyl-CoA dehydrogenase protein from HPB1.1 (query) and acyl-CoA dehydrogenase protein from different strains using the Conserved Domain Database (CDD). Red indicates highly conserved, and blue indicates less conserved. Unaligned residues are shown in grey. Amino acids highlighted in yellow and hash marks (#) correspond with active sites involved in the feature.
